# An innovative model based on machine learning and fuzzy logic for tracking lower limb exercises in stroke patients

**DOI:** 10.1038/s41598-025-90031-1

**Published:** 2025-04-02

**Authors:** Utpal Chandra Das, Ngoc Thien Le, Timporn Vitoonpong, Chalermdej Prapinpairoj, Kawee Anannub, Wasan Akarathanawat, Aurauma Chutinet, Nijasri Charnnarong Suwanwela, Pasu Kaewplung, Surachai Chaitusaney, Watit Benjapolakul

**Affiliations:** 1 Center of Excellence in Artificial Intelligence, Machine Learning and Smart Grid Technology, Department of Electrical Engineering, Faculty of Engineering, Chulalongkorn University, Bangkok, 10330 Thailand; 2 Department of Rehabilitation Medicine, Faculty of Medicine, Chulalongkorn University, Bangkok, 10330 Thailand; 3 Department of Rehabilitation Medicine, King Chulalongkorn Memorial Hospital, The Thai Red Cross Society, Bangkok, 10330 Thailand; 4 Health Service Center, Chulalongkorn University, Bangkok, 10330 Thailand; 5 Division of Neurology, Department of Medicine, Faculty of Medicine, Chulalongkorn University, Bangkok, 10330 Thailand; 6 Chulalongkorn Stroke Center, Chula Neuroscience Center, King Chulalongkorn Memorial Hospital, The Thai Red Cross Society, Bangkok, 10330 Thailand

**Keywords:** Rehabilitation, Lower limb recovery, Fuzzy logic, K-NN model, MediaPipe pose, Biomedical engineering, Stroke

## Abstract

Rehabilitation after a stroke is vital for regaining functional abilities. However, a shortage of rehabilitation professionals leads to many patients with severe disabilities. Traditional rehabilitation methods can be time-consuming and hard to measure for progress. This study introduces an innovative machine learning (ML) approach for lower limb rehabilitation in stroke patients. The proposed methodology integrates two models: a fuzzy logic rule-based system and a K-Nearest Neighbor(K-NN) machine learning model. The rule-based model utilizes the Fugl-Meyer Assessment to evaluate lower limb angles during exercises using a camera without human intervention. The hybrid fuzzy logic-based ML model continuously tracks the desired angle, counts exercise repetitions, and provides real-time feedback on patient progress. Furthermore, it measures the Range of Motion (ROM) for each repetition, presenting a graphical visualization of ROMs for ten repetitions simultaneously. The model facilitates real-time evaluation of rehabilitation progress by clinicians, with the lowest observed error rate of $0.34^\circ$ of angle measurement. The K-NN model assesses rehabilitation exercise accuracy levels, presenting results graphically, with machine learning accuracy rates of $97\%$, $92\%$, and $91\%$ for hip flexion, hip external rotation, and knee extension rehabilitation exercises. Model training utilized data from 30 experienced physical therapists at King Chulalongkorn Memorial Hospital, Bangkok, Thailand, garnering positive evaluations from rehabilitation doctors. The proposed ML-based models offer real-time and prerecorded video capabilities, enabling telerehabilitation applications. This research highlights the potential of ML-based methodologies in stroke rehabilitation to enhance accuracy, efficiency, and patient outcomes.

## Introduction

Stroke is a devastating condition that ranks as the second leading cause of death globally and the primary driver of long-term disability in humans^1^. The effects of stroke can manifest across the body, from the right or left side, upper or lower limbs, or any specific region, depending on the affected brain area. This neurological event imposes a substantial financial burden on healthcare systems and societies around the world. The American Heart Association and American Stroke Association estimate the direct and indirect costs of stroke in the United States alone to be around $\$46$ billion annually^2^. Worldwide, stroke stands as a leading cause of both mortality and disability. According to the World Health Organization, it is the second leading cause of death globally, accounting for approximately $11\%$ of all deaths^3^. Fortunately, the number of stroke survivors is increasing due to an aging population, even as the mortality rate declines. This has led to a significant demand for stroke rehabilitation services, not just for patients, doctors, and clinics, but also for hospitals in need of post-stroke rehabilitation support. The post-stroke treatment aims to mitigate the risk of disease recurrence, yet many stroke survivors experience relapses within the first two years, with an approximate probability of $10\%$. The degree of disability resulting from stroke varies considerably, influenced by factors such as stroke severity, healthcare access, and availability of rehabilitation services. Nevertheless, it is estimated that around $50\%$ to $70\%$ of stroke survivors experience some level of long-term disability^4^.

Stroke survivors often experience impairments in various functions, including cognition, speech, mobility, and sensory abilities. The recovery process encompasses early, late, and residual stages. The rehabilitation plan depends on the extent of brain damage and the patient’s overall health. The Fugl-Meyer Assessment is widely used to evaluate motor recovery, focusing on range of motion and angle measurements^5^. Doctors then create a customized treatment and rehabilitation plan, initially addressing self-care, communication, movement, speech, and cognition, before considering professional skills rehabilitation^6–9^.

Rehabilitation at home following a stroke is a complex and challenging process that requires the guidance of a specialized neurologist. Patients or their caregivers can actively monitor the patient’s condition, such as by measuring blood pressure in individuals with hypertension or managing conditions like diabetes and atherosclerosis that may impact recovery. To facilitate the rehabilitation of post-stroke patients in a home setting, a computer vision-based stroke monitoring application program is necessary. This would enable remote healthcare support from neurologist specialists through a tele-therapy platform that leverages the power of artificial intelligence to monitor stroke rehabilitation progress^10^.

Assessing upper limb recovery through virtual reality (VR) platforms and robotic assistance can be appropriate for post-stroke patients in a home setting^11–13^, However, addressing the rehabilitation needs of the lower limb using VR is complex. Some researchers have explored mirror-based home rehabilitation therapy, and the gait lab presents a potential avenue for lower limb post-stroke rehabilitation, but setting up the gait lab in a home environment is impractical and financially prohibitive for hospitals. Furthermore, as the patient population rapidly increases, effectively scheduling all patients becomes challenging^14–18^.

This study proposes an ML-driven approach to address the issues of lower limb post-stroke rehabilitation. The paper introduces a novel fuzzy logic rule-based hybrid algorithm and a machine learning algorithm for post-stroke rehabilitation exercises, where two distinct methods have been developed to address lower limb rehabilitation problems. In addition, this study also utilizes a K-NN Model for monitoring, tracking, and counting stroke rehabilitation exercises. Through this study, we establish innovative solution of lower limb rehabilitation progress and functional improvement. The key contributions of this study are:

1. We developed a hybrid fuzzy logic-based ML model to monitor, track, and count stroke rehabilitation exercises, as well as extract continuous joint angles and quantify exercise performance,
2. The hybrid model aims to assist clinicians in examinations, motivate patients to exercise at home, and facilitate telerehabilitation, and
3. The proposed model was validated through clinical trials involving lower-limb exercises performed by 30 physical therapists.

The remains of this paper are organized as follows: Section 2 provides an overview of related work. Section 3 details the proposed ML-Driven rehabilitation model for lower limb exercises. Section 4 presents the experimental results of our proposed model. Section 5 gives the discussion. Finally, Section 6 concludes the paper and discusses future work.

## Related work

Stroke rehabilitation has been an active area of research, with a focus on upper limb recovery through virtual reality-based systems and robotic assistance. Robotic devices have demonstrated potential in enhancing the precision and consistency of rehabilitation exercises, while virtual reality platforms have shown promise in engaging patients and providing immersive therapy^19–21^. However, the rehabilitation of the lower limb remains a significant challenge, as the complexity of gait and balance requires specialized equipment and close supervision.

Recent studies have explored the use of machine learning and artificial intelligence techniques to improve the effectiveness of stroke rehabilitation. These approaches aim to enable personalized, adaptive, and autonomous rehabilitation programs that can be implemented in home settings, reducing the burden on healthcare providers and increasing accessibility for patients. One such study proposed an interactive socially assistive robot for personalized post-stroke therapy, where the robot was designed to provide personalized interaction and feedback to patients, addressing the challenge of generating individualized rehabilitation experiences^22,23^. Another study explored the use of auto-adaptive robot-aided therapy, leveraging machine learning techniques to modulate the therapy based on the patient’s state^24–27^.

Building on these previous works, several studies have investigated the application of deep learning and machine learning for stroke rehabilitation. For instance, a comprehensive review outlined the various brain-computer interface-based machine and deep learning algorithms for stroke rehabilitation^28^. Additionally, researchers have explored the use of explainable ML techniques to predict upper limb rehabilitation outcomes in sub-acute stroke patients^29^. A transfer learning framework based on motor imagery rehabilitation has also been proposed for stroke^30^.

Furthermore, deep learning-based approaches have been developed for activity recognition in post-stroke rehabilitation. For example, studies have used comprehensive evaluation of state-of-the-art time-series deep learning models for activity recognition^31^, as well as deep learning-enhanced internet of things for activity recognition in post-stroke rehabilitation^32^. Additionally, researchers have explored the use of gradient-based explainable ML techniques to assess time-series data in post-stroke rehabilitation^33^. Deep learning-based approaches have also been used for human motion decoding in smart walkers for rehabilitation, enabling personalized therapy through adaptive robot-aided therapy^22^. Researchers have further developed deep learning-based systems for detecting and correcting stroke rehabilitation postures in real-time, improving the precision and consistency of rehabilitation exercises^34,35^.

Predictive models utilizing deep learning have been explored for forecasting stroke patients’ recovery in different rehabilitation stages^36^. Additionally, stroke home rehabilitation applications have been developed based on machine learning models, increasing accessibility for patients ?. Lastly, comprehensive reviews on ML-Driven stroke rehabilitation systems and assessment have been conducted, summarizing the state-of-the-art in this domain^37,38^.

## Methodology of ML-driven rehabilitation

**Fig. 1:**
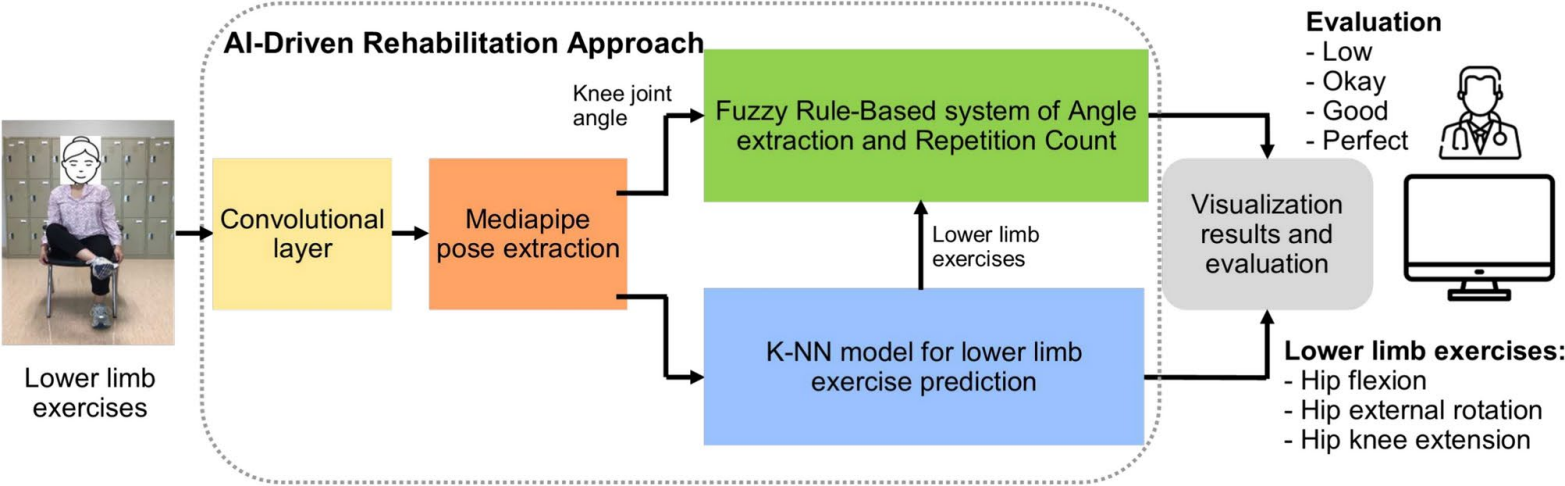
The overall components of our proposed ML-Driven rehabilitation approach for lower limb exercises.

Figure 1 gives the overall components of our proposed ML-Driven rehabilitation approach for lower limb exercises. Unlike others in literature, our approach leverages a hybrid approach combining Convolutional Neural Networks (CNN), Fuzzy rule-based model, and K-Nearest Neighbors (K-NN) algorithms to provide a comprehensive solution for lower limb rehabilitation. We represent the details of each components in the following subsections.

Due to the lack of publicly available video datasets of real stroke patient rehabilitation exercises, the research team collaborated with the Faculty of Medicine at King Chulalongkorn Memorial Hospital to record rehabilitation exercises performed by 30 experienced physical therapists in a clinical setting. Each therapist provided 5 videos demonstrating 3 different lower limb exercises from frontal and side views, which were deemed suitable for evaluating lower limb rehabilitation. The video data were created under the supervision of a rehabilitation doctor. The computational resources utilized in this study included an external camera and a computer with an AMD Ryzen 7 5800H processor, 16 GB of RAM, and an NVIDIA GeForce RTX 3050Ti GPU. Compared to the literature, this video dataset recorded in a clinical environment with experienced therapists is considered more reliable than others in literature for training the proposed ML-Driven rehabilitation model.

### Lower limb recovery exercises

**Fig. 2:**
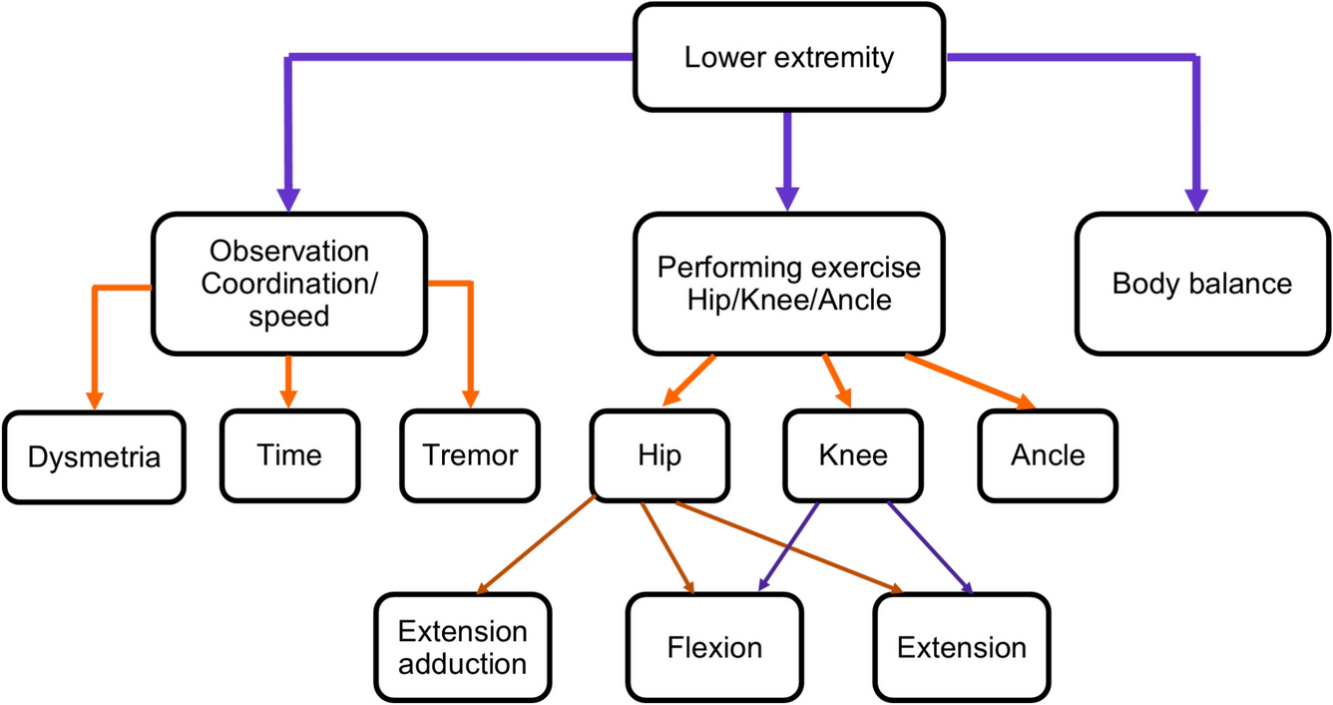
The Fugl-Meyer Assessment (FMA) assessment function of lower limb part for stroke rehabilitation^5^.

Our study aims to propose an ML based approach for lower limb rehabilitation in stroke patients. The research training is divided into two parts. Firstly, neurologist doctors provide exercise instructions and create 30 video datasets with various exercise types for post-stroke patients. Subsequently, the proposed model will be trained using this dataset, initially focusing on describing the human-centric aspects, such as pose, direction, and angle of movement of the patient, followed by the ML-Driven process in the second part of the training. Figure 2 gives the Fugl-Meyer Assessment (FMA) lower limb points^39^. The exercise evaluates the subject’s motor function related to the human brain. It includes testing the patellar, knee flexor, and achilles reflexes. The subject is instructed to flex the hip, knee, and ankle joints to their maximum range of motion while sitting in a chair, usually at the exact moment. Typically, both hip abduction and outward rotation occur simultaneously. To confirm the active flexion of the knee during this motion, the distal tendons of the knee flexor should be palpated. This research also emphasizes the FMA lower limb criteria.

These hip, knee, and ankle point lower limb exercises target specific lower limb muscles and can help improve strength, coordination, and balance. The repetitive movements involved in these exercises can also stimulate the neural connections in the brain, helping to rewire and enhance the motor pathways damaged by the stroke. Regular exercise can also help to improve circulation and oxygenation to the brain, which can aid in the recovery process and support overall brain health.

#### Hip flexion

**Fig. 3:**
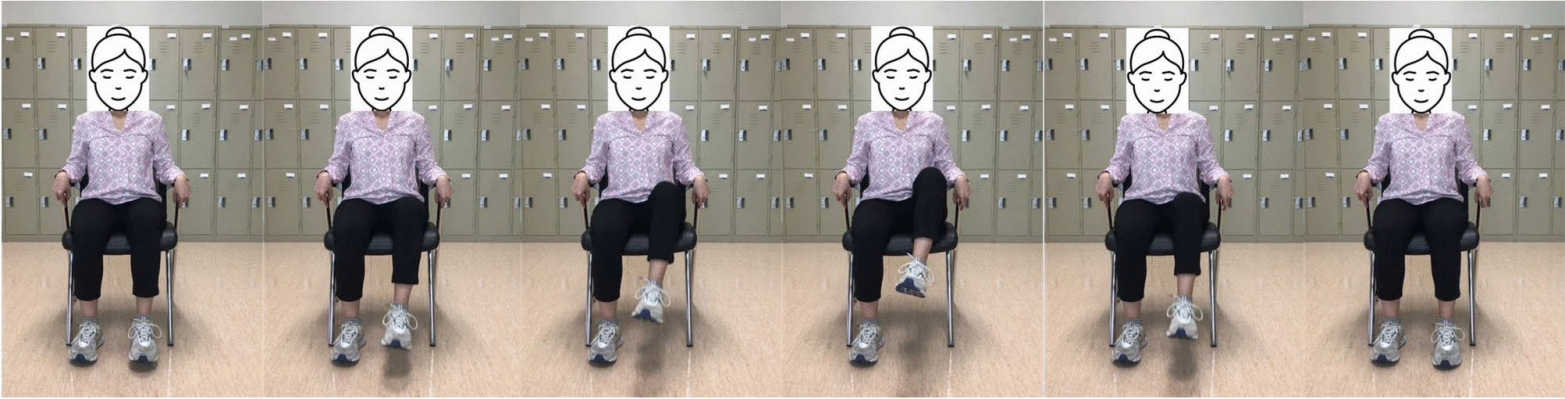
From left to right sub-figures: Example hip flexion exercise on left leg.

Patients with restricted mobility benefit significantly from this leg exercise since they can use their arms to support their legs. To start this exercise, use the patient’s hands to lift their affected leg into the chest. Hold there for a second before slowly letting the leg back down. Repeat on the other leg. This type of exercise targets the hip flexor muscles and helps to improve their strength and flexibility. These muscles can aid in walking and rising from a chair. These exercise positions are illustrated in Fig. 3.

#### Hip external rotation

**Fig. 4:**
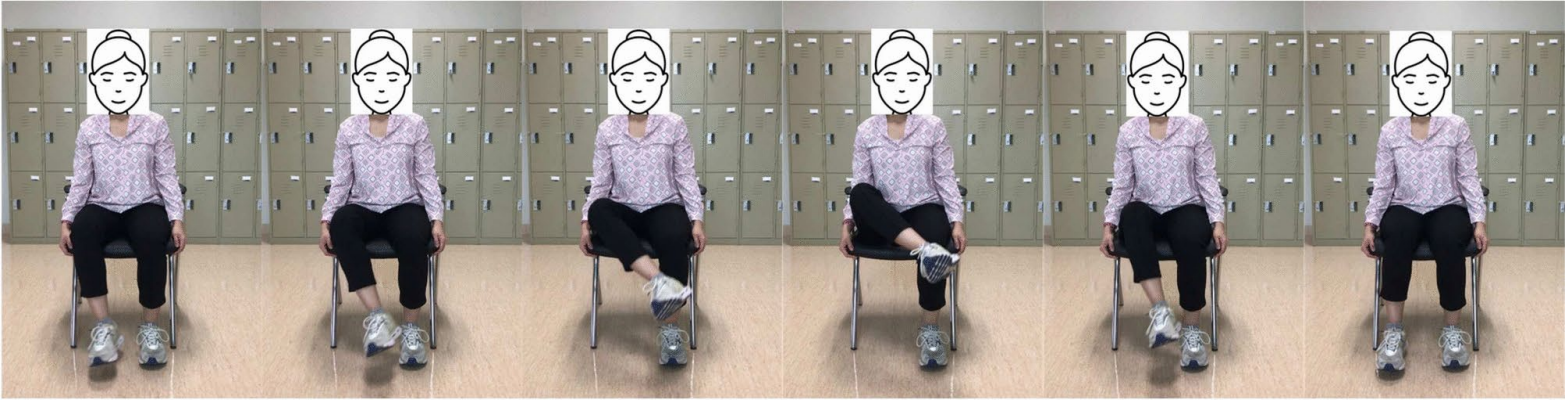
From left to right sub-figures: Example hip external rotation exercise on right leg.

Compared to the first exercise, hip external rotation is more complex but still suitable for patients with limited mobility. To facilitate movement, start by putting a towel beneath the troubled foot. Then, use the subject hands to assist the affected leg and slide the foot toward the mid-line. Then, push the patient’s leg outwards, using hands for assistance if it is essential. This type of exercise targets the muscles that rotate the hip outward, which can help improve mobility and stability in this joint. This can be particularly important for post-stroke patients who experience difficulty with weight-bearing activities due to hip weakness. These exercise positions are illustrated in Fig. 4.

#### Knee extension

**Fig. 5:**
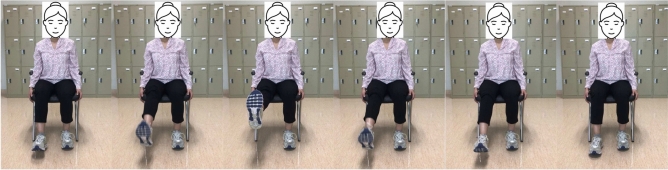
From left to right sub-figures: Example knee extension exercise on right leg.

For stroke patients, a more challenging leg exercise is knee extension. To begin, it necessitates considerable leg movement. It is starting the exercise while seated. Next, straighten the left knee and stretch the leg parallel to the ground. Instead of locking out the knee, try to keep it supple. After that, slowly lower the foot to the ground. After that, repeat with the right leg, switching between the right and left legs. This type of exercise focuses on strengthening the muscles that control knee extension, which can help to improve walking speed and stability. These exercise positions are illustrated in Fig. 5.

**Table 1:** Summary three lower limb exercises used for stroke patient in our study.

Motion	Starting position	Stabilization	End range
Hip Flexion (Fig. 3)	Sitting in a chair hips, and knees are in $90^{\circ }$ flexion, $0^{\circ }$ of abduction. Lift up the leg slowly as much as possible, keeping the knee and ankle aligned. Ensure the chair is stable and secured to prevent any risk of falls. If necessary, provide assistive devices such as a gait belt.	Flex the hip joint to bring the knee towards the chest in a slow and controlled manner.	Once maximum hip flexion is achieved without causing discomfort, hold the position for a few seconds to improve range of motion and muscle activation.
Hip External Rotation (Fig. 4)	Sitting in a chair position with hips and knees in $90^{\circ }$ flexion, $0^{\circ }$ of abduction, and adduction concerning the ankle position.	Knee bending and rising ankle movement of the hip.	The knee position is outward and the ankle is the highest position it can reach above.
Knee Extension (Fig. 5)	Sitting in a chair, the hips and knees are in $90^{\circ }$-flexion.	Throughout, the focus is on stabilizing the pelvis and maintaining proper alignment.	The goal is to extend the knee to its full range without strain or hyperextension, emphasizing controlled movement and engagement of the quadriceps.

Table 1 describes the three exercises and how to perform them. This research aims to create an ML-based model for aiding in the clinical diagnosis and home rehabilitation of post-stroke patients. We checked our model in both home and clinical or hospital-based environments.

### MediaPipe pose

**Fig. 6:**
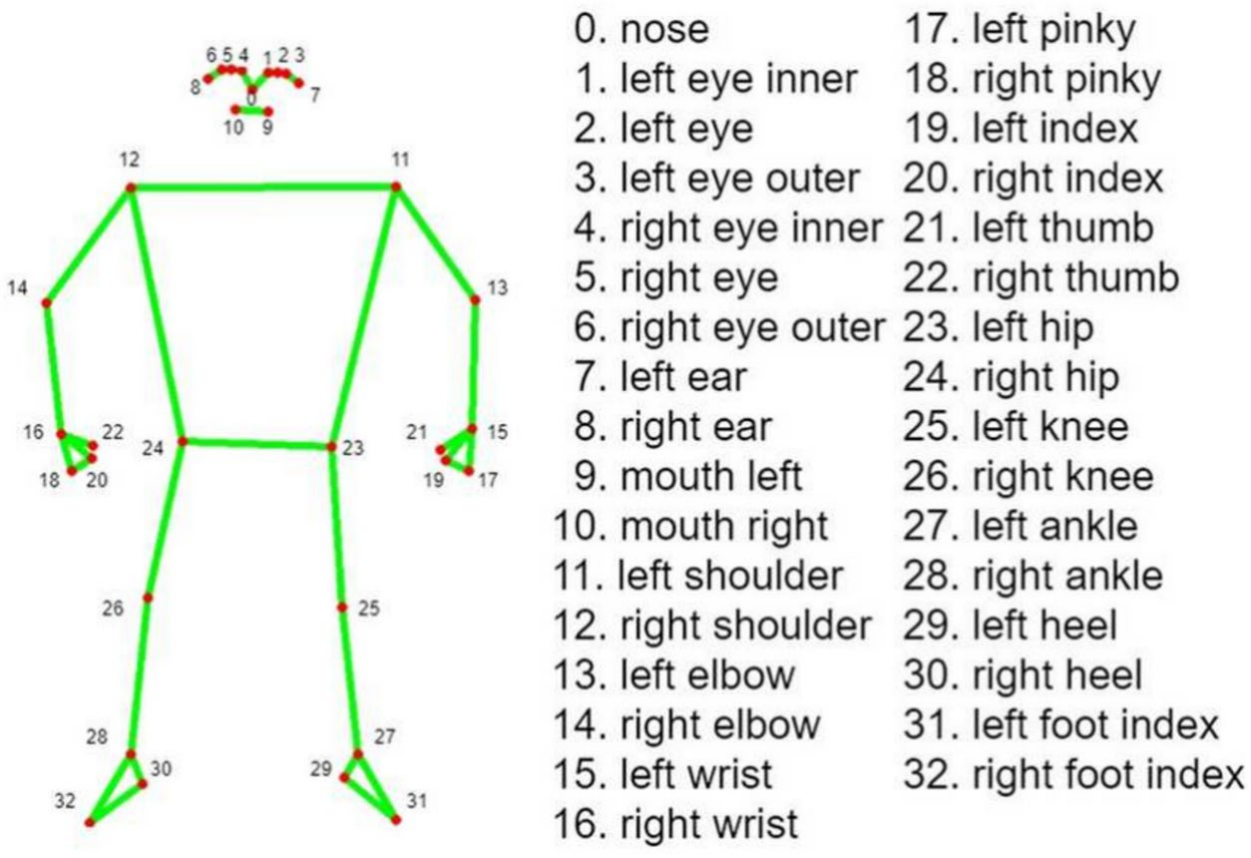
Demonstration of 33 landmarks detected on the human body using MediaPipe^40^.

MediaPipe Pose is an ML solution for high-fidelity body pose tracking, inferring 33 3D landmarks and background segmentation masks on the whole body from RGB video frames utilizing Blaze Pose research that also powers the ML Kit Pose Detection API. Current state-of-the-art approaches rely primarily on powerful desktop environments for inference. In contrast, the proposed method achieves real-time performance on most modern mobile phones, desktops/laptops, python, and the web. The MediaPipe Blaze Pose model is based on the COCO topology, consisting of 33 landmarks across the torso, arms, legs, and face, as presented in Fig. 6. However, the COCO key points only localize to the ankle and wrist points, lacking scale and orientation information for hands and feet, which is vital for practical applications like fitness and dance. Including more key points is crucial for the subsequent application of domain-specific pose estimation models, like those for hands, faces, or feet. With BlazePose, the authors present a new topology of 33 human body key points, a superset of COCO, BlazeFace, and BlazePalm topologies. This allows them to determine body semantics from pose prediction alone, consistent with face and hand models.

For pose estimation, the two-step detector-tracker ML pipeline is utilized. This pipeline first locates the pose region of interest (ROI) within the frame. The tracker subsequently predicts all 33 pose key points from this ROI. Notably, the detector is run only on the first frame for video use cases. The pose estimation component of the pipeline predicts the location of all 33 key points with three degrees of freedom each (x, y location, and visibility) plus the two virtual alignment key points described above. The model uses a regression approach supervised by a combined heat map/offset prediction of all key points^41^. Specifically, during training, the pipeline first employs a heatmap and offsets the loss to train the center and left tower of the network. It then removes the heatmap output and trains the regression encoder, thus effectively using the heatmap to supervise a lightweight embedding.

It is important to note that exercises should be tailored to each individual’s specific needs and abilities. Therefore, it is crucial to stress that these exercises should be performed under the guidance of a physical therapist. Moreover, the therapist will be able to oversee the patient’s progress and modify the treatment plan as necessary.

### Training of proposed models

In this research, we implemented two separate machine learning models: one is K-NN, and the other is the hybrid rule-based model. In the hybrid model, the CNN model draws the human skeleton and shows the joint points. Fuzzy logic rules are applied to extract the specific joint angle and count the exercise of the hybrid model. The k-NN model visualizes the exercise graph based on the train data set and counts the perfect exercise. A predefined machine learning model is used for skeleton detection and is an input in both cases.

**Fig. 7:**
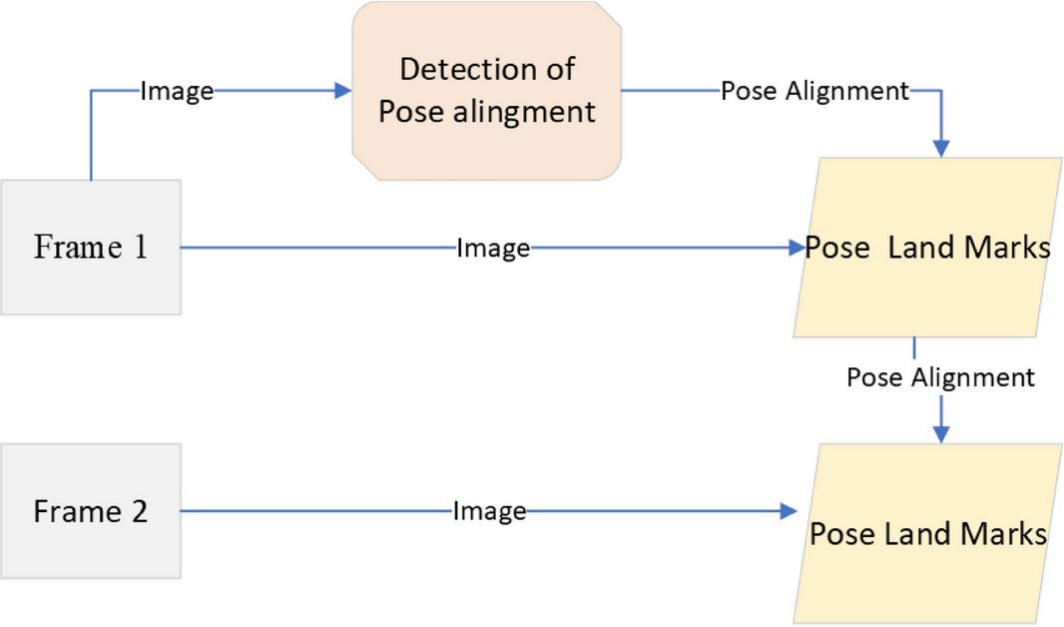
Model Input frame to pose landmarks.

**Fig. 8:**
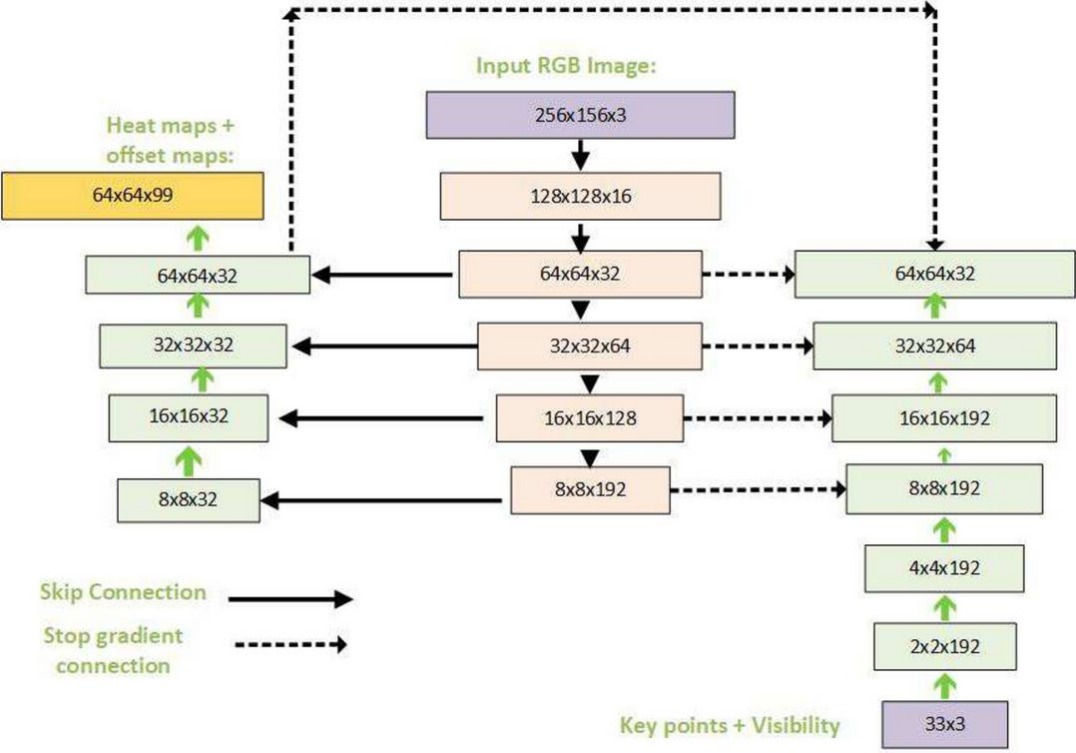
Tracking network architecture: regression with heatmap supervision.

From the video or real-time human skeleton pose tracking system, a general framework is shown in Fig. 7. MediaPipe ML algorithm tracks the pose landmarks in this way. The skeleton’s media pipe draw depends on the input RGB image and generates the heatmaps of the key point locations. The work of crucial point visibility can be described in this way: the input frame detects pose alignment with heatmaps, and pose landmarks detect pose alignment. The network architecture of the regression with heatmap supervision is shown in Fig. 8. The 8-by-8 frame size to 64-by-64 frame size image is interconnected with heatmap input RGB and key point visibility.

The machine learning-based model output is accuracy, and it visualizes the graph of accuracy that it learns from the accurate video dataset created for this research study. For training purposes, the dataset was used from King Chulalongkorn Memorial Hospital, consisting of 30 subjects performing post-rehabilitation exercises, all confirmed by a neurologist and a physiotherapist. Here, the data is used to train and evaluate the K-NN algorithm through the training process. Then, the angle of every video data is extracted. Later, it adjusted with the extracting angle and FMA logic. The hybrid noble model for the automatic angle measurement of the exercise in the desired point moving part according to the FMA method, or we can set our own defined rule for the patient, which was not previously done using any ML model. We employed MediaPipe to draw the human skeleton over the patient’s body. We chose the Blaze Pose Lite MediaPipe, which has a 33 key point topology, for its updated version and the additional information it provides, including the number of joints and critical points, which aids in diagnosing the patient. This model can measure any specific joint angle from 33 joint points and gives the status of the angle’s changing while counting the reps. Our approach enables doctors to monitor their patients’ progress efficiently without needing a stopwatch or assistance counting and tracking the rehabilitation exercises. Furthermore, with scale and rotation, additional information, such as hands, faces, and feet, helps diagnose.

#### Fuzzy rule-based model

**Fig. 9:**
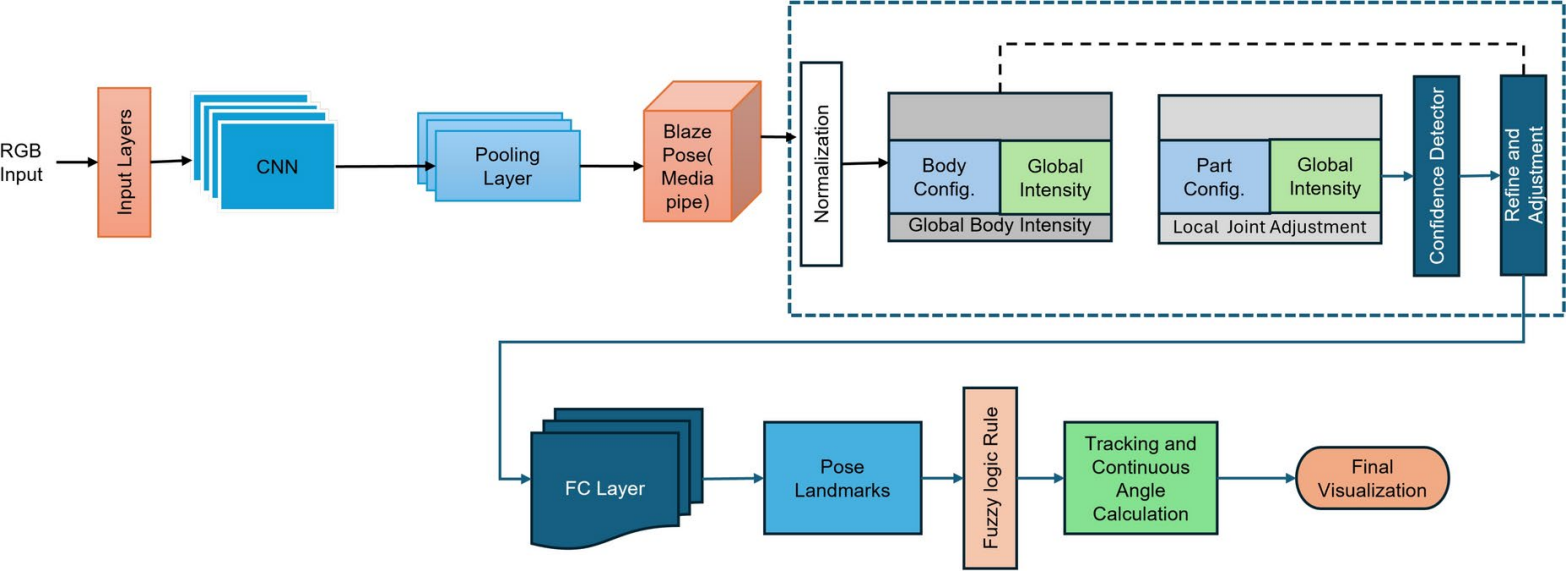
Proposed model framework for human pose refinement and angle calculation process.

**Fig. 10:**
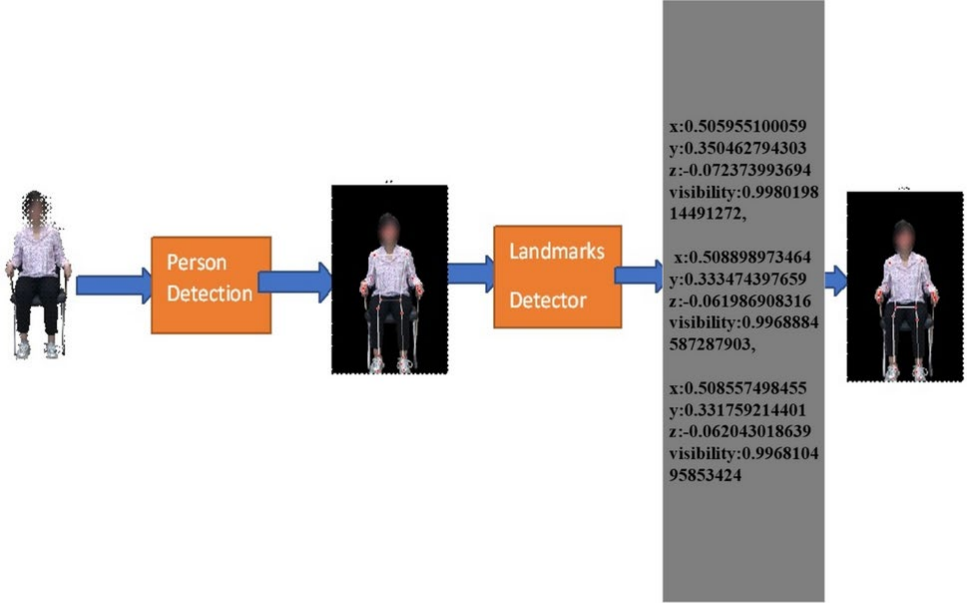
Human person drawing the MediaPipe landmark and extracting the landmarks point.

The summary of the proposed fuzzy rule-based hybrid ML model is described in this section. The proposed model learns the human body’s skeleton position, which are visualized in Figs. 9 and 10. With the movement of the body, the skeleton will also move. It will also track how many exercises have been completed and display that information on the screen. The machine learning part of this, which we already discussed above, is that the heat map generation from the image frame and pose alignment draws the body’s skeleton. Moreover, counting the exercises performed is also very important because these are compared with the medicine dose. Both of these things are addressed in the hybrid model. Detecting the whole-body skeleton in this coding screenshot uses MediaPipe computer graphics. The first step is to call the media pipe pose and change the screen image to RGB. Then, detection is made, and the detection is drawn. The user can press a key to stop the process. The human body skeleton’s key points, or joint structures, are extracted using MediaPipe computer graphics, and 33 key points are identified. The landmarks are used to identify the 33 key point joints and the key points are determined based on the researchers’ requirements. A hybrid model architecture is used for the post-rehabilitation phase. The data is one for machine learning pose estimation and the other for proper angle representation.

The 2D system has a two-point X axis, described from left to right, and the Y axis is the point from bottom to top. For this model to calculate the joint angle movement, we must measure the 3D skeleton point. The 3D space point is used to measure the point of the media pipe skeleton. X, Y, and Z, and after that, it will be visible. We can also call it visualization or detecting skeleton points. Figure 10 shows the example detection of the human skeleton from the human body through the proposed method and how it extracts the landmark points of body joints.

1 $$\begin{aligned} ax+by+cz+d=0 \end{aligned}$$

Here, we give our lower limb part 3D value with visibility. Later, we reduced our 3D points to 2D points. In Eq. (1), we have the *x*, *y*, and *z* for every joint point. *x*, *y*, and *z* represent the positions of the point along the x-axis, y-axis, and z-axis. These three points represent the 3D visualization of each floating-point joint point. Visibility is the confidence level in showing the point on the screen. It is determined by 0 and 1; above 0.9, it has high confidence in visibility. Equation (1) represents the plane of 3D space, where *a*, *b*, and *c* are the coefficients of *x*, *y*, and *z*, and d is a constant that determines the plane’s position. It also represents the distance from the origin.For 3D joints, our model output and visibility are as follows.

$$\begin{aligned} \text {Left Hip:} {\left\{ \begin{array}{ll}x = 0.5340\\ y = 0.4754\\ z = -0.0036\\ visibility = 0.9999\end{array}\right. } \\ \text {Left Knee:} {\left\{ \begin{array}{ll}x=0.5408\\ y=0.5138\\ z=-0.1875\\ visibility=0.9978\end{array}\right. } \\ \text {Left Ankle:} {\left\{ \begin{array}{ll}x=0.5396\\ y=0.7088\\ z=-0.1298\\ visibility=0.9969\end{array}\right. } \end{aligned}$$

The above shows the value of *x*, *y*, and *z* for the particular landmark points of the left hip, left knee, and left ankle. 3D key point representation of the hip, knee, and ankle are the most important parts of our lower limb exercise. The 3D representation of key points in the representation is mathematically shown above for the left hip, left knee, and left ankle in their 3D positions.

We need to calculate the angle for the most important part of the rule-based model. To calculate the angle, we must set the rules. When we define the rules in Python and set the rules for determining the joint angle when we perform the exercise, we need the three-point starting point, mid-point, and end point. When the starting and ending points of the mid-point are changed, the degree of angle of the mid-point changes accordingly. The mathematical rule set for angle also describes the rules for radian and angle. We convert the 3D point into a 2D point for ease of calculation. The joint angle was calculated as the difference in angle between two adjacent segments’ longitudinal axes. These segments comprise three points in the 2D space: the beginning, the center, and the end. The neighboring segments for the knee joint angle were the lower limb and the crus, respectively, from knee to ankle. The measurements made for the knee and hip joints in this investigation.

Figure 9 shows the proposed method framework for identifying human landmarks. In this case, the model’s input is the RGB frame, which is resized with the convolutional and pooling layers. The base network predicts the pose with 33 landmark points.

**Fig. 11:**
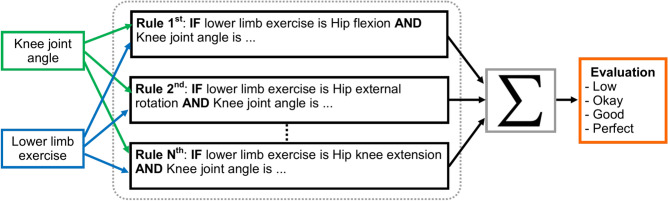
Flowchart of proposed fuzzy rule-based model for lower limb rehabilitation.

2 $$\begin{aligned} \left[ \begin{array}{l} X_{C K 1} \\ Y_{C K 1} \\ Z_{C K 1} \end{array}\right] =R\left[ \begin{array}{l} X_{K 1} \\ Y_{K 1} \\ Z_{K 1} \end{array}\right] +T \end{aligned}$$

3 $$\begin{aligned} \bigg \lceil u=\left( \frac{x}{z}\right) * f_x+c_x v=\left( \frac{y}{z}\right) * f_y+c_y\bigg \rceil \end{aligned}$$

In Fig. 11, the blue arrow indicates the step-by-step in the flowchart. The blue arrow indicates the yes condition, and the black arrow indicates the no logic condition. In Equation (2), the model working is derived using mathematical rules to extract the joint representation angle.

In Eq. (2), K1 coordinate system

$$\begin{aligned} \left[ \begin{array}{l} X_{C K 1} \\ Y_{C K 1} \\ Z_{C K 1} \end{array}\right] \end{aligned}$$

the coordinates all refer to the same place in the coordinate CK1 system. The 3x3 rotation matrix R describes the orientation of the CK1 coordinate system with the K1 coordinate system. The unit vectors of the CK1 coordinate system expressed in the K1 coordinate system are shown in the columns of R. All refer to the same place in the CK1 coordinate system. The orientation of the CK1 coordinate system in relation to the K1 coordinate system is described by the 3x3 rotation matrix R. The unit vectors of the CK1 coordinate system expressed in the K1 coordinate system are shown in the columns of R.

The position of the CK1 coordinate system with the K1 coordinate system is indicated by the translation vector $[T_x,T_y,T_z]$.

It reflects the displacement in the $K_1$ coordinate system from the origin of $K_1$ to the origin of $CK_1$. Equation (3) represents the values of $u$ and $v$, denoting the point’s horizontal and vertical coordinates in the picture plane. The camera’s focal lengths along the horizontal and vertical axes are $f_x$ and $f_y$ respectively. In terms of pixels, it indicates the separation between the image plane and the camera’s center.

The intersection of the camera’s optical axis and the picture plane is the primary point of the camera, and its coordinates are $(c_x, c_y)$.

The vector in pixel coordinates $\textbf{u}=[u_x,u_y]$ denotes the direction from the camera’s center to the projected 3D point in the image plane. It represents the angle calculation derived through the vector. The vector denoted by $\textbf{v} = [v_{x}, v_y]$ points from the camera’s center to an image-plane reference point, such as the main or image’s center.

The symbol $\cdot$ denotes the dot product. $|\textbf{u}|$ and $|\textbf{v}|$ represent the magnitudes of the two vectors. The result of multiplying the lengths of the two vectors by the cosine of the angle that separates them $\theta$ is denoted as $\mathbf {u \cdot v} = |\textbf{u}| \cdot |\textbf{v}| \cdot \cos {\theta }$.

#### Machine learning representation

The appropriate samples should be collected for the training set to build a good classifier for each terminal state of each exercise (e.g., “up” and “down” positions for rehabilitation exercise). Collected samples must cover camera angles, environmental conditions, body shapes, and exercise variations. This work creates the training video datasets by following the lower limb exercises. The stroke recovery exercises focus on the legs to help affected patients improve or enhance their gait (way of walking) and balance. This type of training of the legs can also help reduce the risk of falling, which is a priority for all stroke survivors. This type of exercise is also very effective for elderly people. The hip flexion, external hip rotation, and knee extension exercises are described and presented in Figs. 3, 4 and 5.

**Fig. 12:**
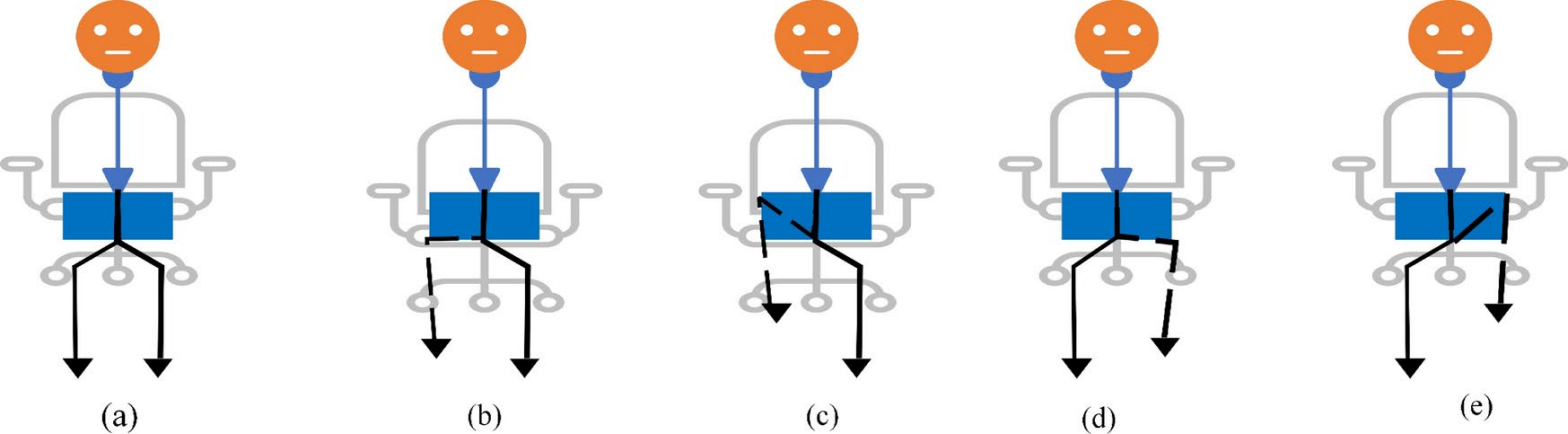
Training process angle calculation among HIP, KNEE, and ANKLE points. (**a**) sitting in the normal pose (**b**) start moving of left leg (**c**) target position (**d**) start moving the right leg (**e**) right leg targeted position.

**Fig. 13:**
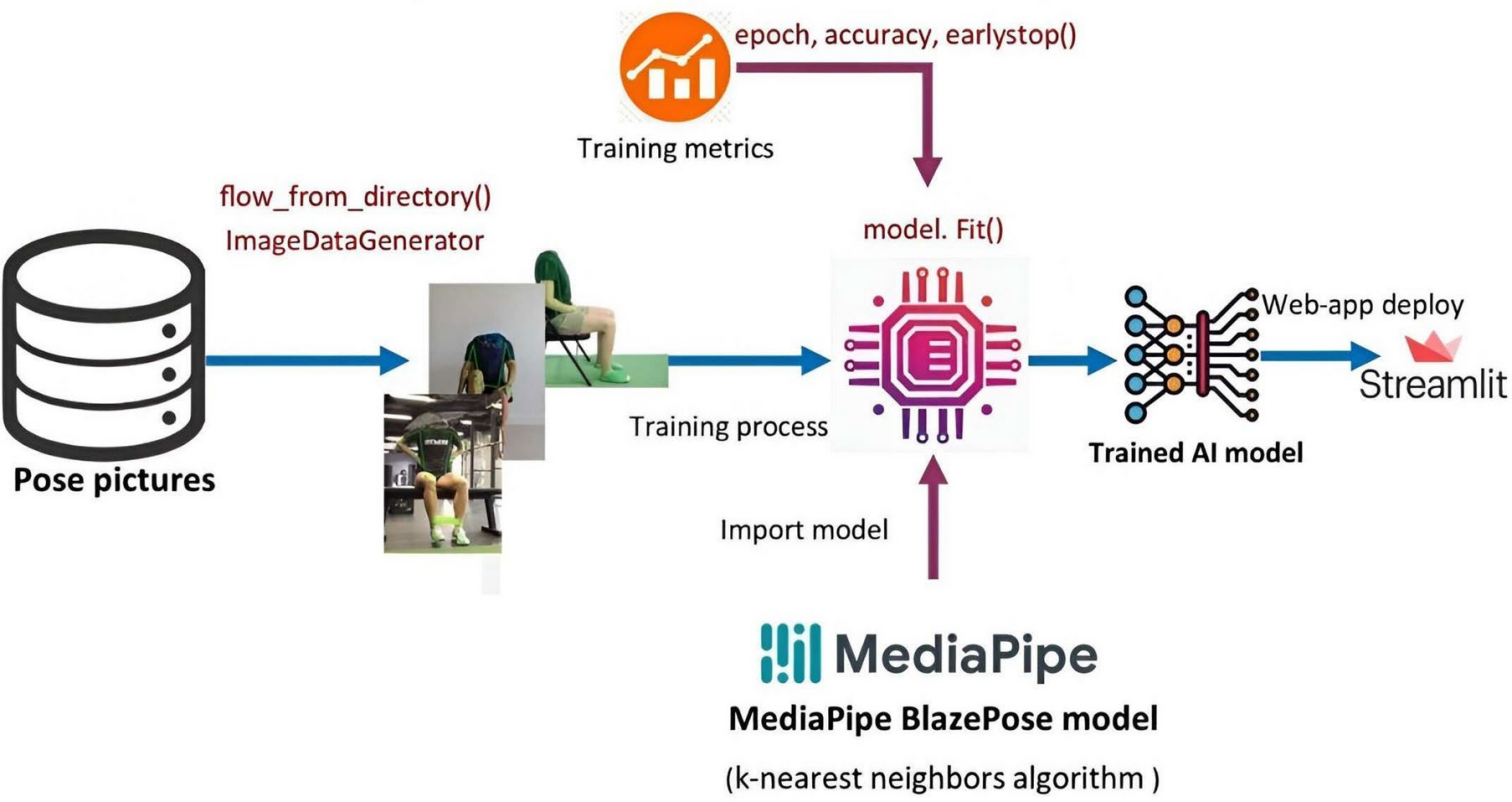
The overall pipeline process of training K-NN machine learning model.

Figure 12 shows the hip flexion exercise step by step from (a) to (e). The overall stroke rehabilitation monitoring processes are evaluated, as shown in Fig. 13, based on the MediaPipe Pose model from Google AI. Google Colab loads the MediaPipe Pose model into the Keras platform to support training ML models. All the training image datasets are uploaded to Google Drive to accelerate the training pipeline and applied to the 33 human body key points. Next, the key-pointed images are loaded to the K-nearest neighbors (K-NN) algorithm to classify the rehabilitation activities. Then, the trained ML model is implemented as the backend engine for the web apps of the stroke system via the platform to build webapps or even mobile applications. The mathematical equation of k-NN is given in Eqs. (4) and (5).

4 $$\begin{aligned} \begin{array}{r} d\left( x, x^{\prime }\right) =\sqrt{\left( x_1-x_1^{\prime }\right) ^2+\cdots +\left( x_n-x_n^{\prime }\right) ^2} \\ \end{array} \end{aligned}$$

5 $$\begin{aligned} \begin{array}{r} P(y = \mid j \mid \mid X=x)=\frac{1}{K} \sum _{i \in A} I\left( \left( y^{(i)}=j\right) \right. \end{array} \end{aligned}$$

Equation (4) represents the Euclidean distance between two *n*-dimensional points, *x* and $x'$. Now $x = (x_1, x_2, \ldots , x_n)$ represents an *n*-dimensional point with *n* components, while $x'=(x_1',\ldots ,x_n')$ represents another *n*-dimensional point with *n* components. The expression $(x_i - x_i')$ represents the difference between the *i*-th component *x* and the *i*-th component of $x'$. The expression $(x_i - x_i')^2$ represents the square of the difference between the *i*-th component of *x* and the *i*-th component of $x'$.

Equation (5) represents the conditional probability of a discrete random variable *y* taking the value *j*, given that another random variable *X* takes the value $\kappa$. In this equation, $P(y=|j||X= x)$ represents the conditional probability of $y=|j|$ given $X=x$. *K* is a normalizing constant that ensures that the probabilities sum up to 1. $\sum _{i\in A}$ represents the sum over all *i* in set *A*, a subset of the index set of the data points. $I((\hat{y}^{(i)}=j))$ is an indicator function that evaluates to 1 if the *i*-th data point has the label $\hat{y}^{(i)}=j$, and 0 otherwise.

The K-NN model training pipeline process is done via the Google Colab environment. The model’s accuracy values are shown in Table 2 in the experimental results section. The accuracy is calculated as the total number of recognized exercises by the trained K-NN model over the total number of exercises done. More datasets of those exercises can be collected to retrain the model to improve the accuracy performances.

#### Vector format of angle extraction

Equation (6) gives the vector format of angle extraction. The $\varvec{a}$ and $\varvec{b}$ represent two neighboring segments with vectors $\varvec{a}$ and $\varvec{b}$, where the angle between $\varvec{a}$ and $\varvec{b}$ is equal to $\varvec{\theta }$. The dot product is defined as the product of the vectors’ magnitudes multiplied by the cosine of the angle between them.

6 $$\begin{aligned} \begin{aligned}&{\varvec{a} \cdot \varvec{b} } =|\varvec{a}||\varvec{b}| \cos (\theta ) \\&\cos (\theta )=(\varvec{a} \cdot \varvec{b}) /|\varvec{a}||\varvec{b}| \\&\varvec{\theta }=\arccos \frac{\varvec{a} \cdot \varvec{b}}{|\varvec{a}||\varvec{b}|} \\ \end{aligned} \end{aligned}$$

#### Geometry format of angle extraction

We used geometric calculation to determine how accurate the angle prediction is, based on the hybrid fuzzy rule-based ML model. We later validated this angle with the mathematical calculation of the visualization angle. Here, the desired angle is cos A, which is from Euclidean geometry. Through Euclidean geometry, it is possible to figure out the angle joint between two rays. In this case, it is assumed that one ray is AB, and another is AC, and the distance BC determines the degree of the angle formed by the two rays. We can define AB = c, AC = b, and BC = a. The geometric equation is given below.

7 $$\begin{aligned} \begin{aligned}&\cos (A) = \frac{ b^2 + c^2 -a^2 }{2bc} \end{aligned} \end{aligned}$$

Equation (7) describes the relationship between the cosine of an angle $A$ (equivalent to $\theta$ in a triangle) and the lengths of its sides. In this equation, a, b, and c are the lengths of the sides of the triangle. A is the angle opposite to the side of length a. cos(A) is the cosine of angle A.

#### Statistical analysis

We measured knee and hip joint angles at the starting and end range of the exercise using our hybrid rule-based model. A total of 30 physical therapists performed hip flexion, knee extension, and hip external rotation with 10 repetitions separately for each leg. The mean range of motion was $40.20^\circ$ degrees, with a median of $40^\circ$ degrees and a standard deviation of $16.721^\circ$ degrees, calculated for each joint with $95\%$ confidence intervals. To assess the reliability of the measurements, we analyzed variance (ANOVA) using a linear mixed effects model in Python. The model was fitted with the formula range of motion group’. The resulting intraclass correlation coefficient (ICC) was 0.99, indicating perfect group agreement. Additionally, using G*Power (Version 3.1), a statistical power analyses, we calculated the sample size needed to detect a significance level of 0.05 or less with a power of 0.96 based on the correlation coefficient of the range of motion. The calculated sample size was 600. Assuming 10 independent trials per exercise for each leg, 30 subjects were required. Statistical analysis was performed using Python.

## Experimental results

### Trained K-NN model

Table 2 shows the summary of the precision and recall values of the trained K-NN model. The highest output accuracy is in the hip flexion exercise, $97\%$; hip external rotation output accuracy is $92\%$, and knee extension is $91\%$. The hip flexion exercise has the highest accuracy. The accuracy is determined by multiplying the total number of hip flexion exercises recognized by the model by the total number of hip flexion exercises performed by the patient. More datasets of those exercises can be collected to retrain the model and improve external hip rotation and knee extension accuracy scores.

**Table 2:** The trained K-NN model for prediction the lower limb exercises and its accuracy.

Trained model	Rehabilitation exercise	Accuracy (%)
K-NN	Lower limb, Hip flexion	97
K-NN	Lower limb, hip external rotation	92
K-NN	Lower limb, knee extension	91

Table 3 shows a comparative analysis of our trained K-NN model with existing machine learning models in literature. Remark that the training dataset is different because there are no existing public video datasets for lower limb rehabilitation. In our proposed model, the real-time graphical representation is also shown. The authors in^42^ used a different technique for detecting the activities, and the number of detected activities was seven. In the first method, the authors used the K-NN model to detect, the percentage is $86.1\%$, which is good. Another algorithm the authors used is random forest; this algorithm’s percentage of accurately detecting the activities is only $80.6\%$, which is lower than the our K-NN model. Through the LSTM (long short-term memory) algorithm, the accuracy is lower than the K-NN algorithm but higher than the random forest model; the accuracy is $82\%$. Another study^43^ used the algorithm Multifused + Accumulated HMM (Hidden Markov Model), and their output accuracy is $72\%$. In conclusion, we used the K-NN algorithm for the proposed ML-Driven model. The output accuracy of the proposed K-NN model is the highest and better than this existing model. The training model and test, as well as the expertise of the neurologist and rehabilitation experts at the King Chulalongkorn Memorial Hospital, have been verified. The doctor can compare it with the FMA scoring method according to patient performance.

**Fig. 14:**
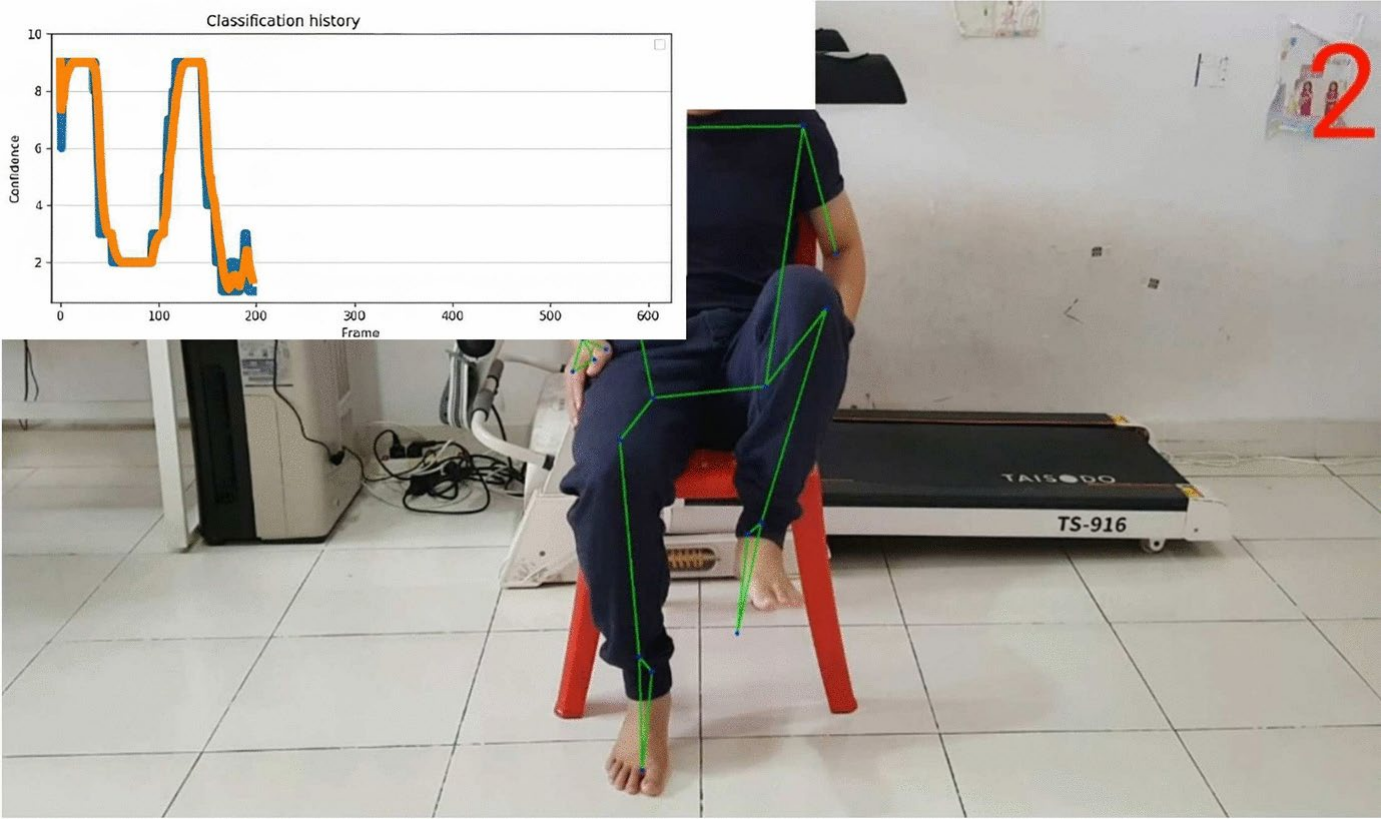
Result visualization of hip flexion prediction given by our K-NN model. The confidence level of classification is vary from 0 to 10.

Figure 14 depicts an example of the visualization of our trained K-NN model, where we can see the prediction result given by our with hip flexion exercises and show the complete range of motion from the patient’s starting position by the classification graph. The graph also can be collected to allows the doctor to see the difference in performance between many stroke patients. The neurologist can easily compare progress to previous time performance for stroke patients. It can also be used as a trajectory movement.

**Table 3:** The accuracy comparison between our trained K-NN model with existing models.

Study	Algorithm	Number of subjects	Number of poses exercises per subject	Accuracy
Ince et al.^42^	K-NN	10 people	7 (human poses)	$86\%$
Ince et al.^42^	LSTM	10 people	7 (human pose)	$82\%$
Ince et al.^42^	Radom Forest	10 people	7 (human pose)	$80.6\%$
Jalal et al.^39^	Multi-fused features; Accumulated HMM	15 people	15 (daily routine activities)	$93.3\%$
Li et al.^43^	CNN	40 patients with chronic stroke	1 (Single leg-stance)	$84\%$
Our model	K-NN	30 physical therapists	3 (Lower limb recovery)	$93.3\%$

**Table 4:** Predicted and actual lower limb rehabilitation exercise angle.

Subject body posture analysis	Rehabilitation exercise type	Degree of trajectory condition	Body part	Predicted Angle by ML model	Actual angle by mathematical calculation	Error
Sitting on the Chair starting position	Ready to do	No	Lower Limb	177.32°	180°	− 2.68°
Sitting on the chair starting position	Ready to do	No	Lower Limb	135.95°	123°	4.95°
Performing Left leg Exercise	HIP Flexion	Greater than 160° and smaller than 15°	Lower limb Right leg Normal condition	151.52°	148°	3.52°
Performing Right leg Exercise	HIP Flexion	Greater than 160 °and Smaller than 15°	Lower limb left leg Normal Condition	141.35°	142°	− 0.65°
Performing Left leg Exercise	HIP Flexion	Greater than 160°and smaller than 15°	Lower limb left leg performing while complete the condition	12.49°	12.84°	− 0.34°
Performing Right leg Exercise	Hip Flexion	Greater than 160°and smaller than 15°	Lower limb right leg performing while complete the condition	3.51°	6.1°	− 2.59°
Performing Left leg Exercise	Hip External Rotation	Greater than 150°and Smaller than 30°	Lower limb left leg performing while complete the condition	45°	46°	− 1°
Performing Left leg Exercise	Hip External Rotation	Greater than 150 °and Smaller than 30°	Lower limb right leg normal condition	90°	93°	− 3°
Performing Left leg Exercise	Knee Extension	Greater than 160° Smaller than 95°	Lower limb left leg performing while complete the condition	85°	88°	− 3°

### Trained fuzzy rule-based model

The proposed fuzzy rule-based model first step is to determine the angle of the desired joint. We set the rule from the extracted video supervised by rehabilitation and neurologist specialists. In the rule-based model, we also visualize the angle of the joints when performing the exercise. The angle of $\theta$ is determined by the knee joint point, which corresponds to the hip and ankle joint points. The angle is calculated from $0^\circ$ to $180^\circ$. With our knee argument, if the joint meets the mathematical condition rules, it will count as one. The doctor can see the joint angle in the rule-based model. While traditionally, doctors or therapists may measure biofeedback starting from the 0° or neutral position, Biofeedback is an innovative approach to stroke rehabilitation that aims to improve patient motor function. To determine the different statuses here, we set up specific fuzzy logic rules that start from 180° in the hybrid model. With the different subject age ranges and body compatibility, the model can be observed with the angle changing and varying to check the angled variety, dose-finding, and stroke patient care. Later, we developed another fuzzy logic to diagnose the patient’s movement more efficiently. We also verify our system with our subject video and real-time subject checking.

Algorithm 1 shows the pseudo code of the status changing of hip flexion exercise. For the hip flexion, if $\theta$ is greater than $130^\circ$, it means the “starting position (ST)”; when it is less than or equal $130^\circ$ and greater than $100^\circ$, it reaches the “Low” status. In the range of less than or equal $100^\circ$ and greater than $60^\circ$, it reaches the status of “Okay”; within the range of greater than $15^\circ$ and less than or queal $60^\circ$, it is “Good”, and lastly, when it is less than or equal $15^\circ$, its status changes to “Perfect”. Algorithm 1 elucidates the rules-based output devised for rehabilitation exercises through comprehension of the FMA and extensive consultations with rehabilitation experts.

**Fig. 15:**
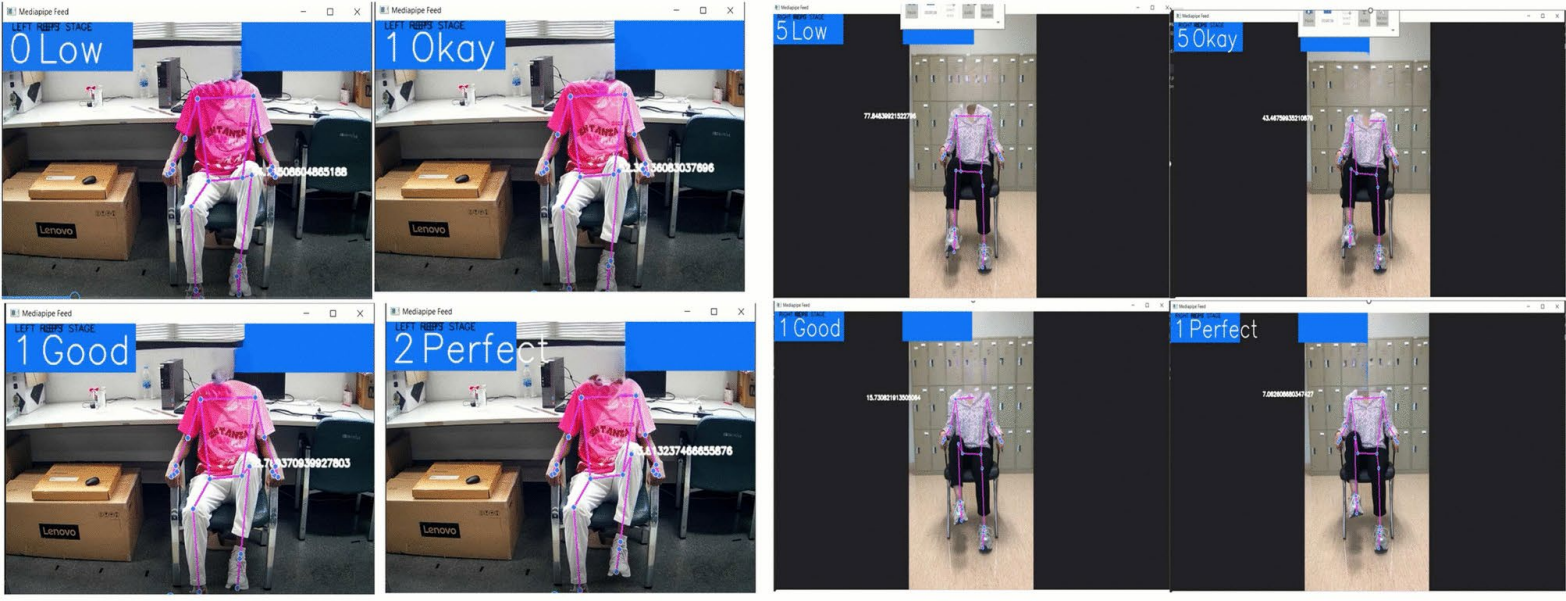
Four status results of hip flexion exercise from the real-time and recorded video.

Figure15 shows the output result of Algorithm 1 to evaluate hip flexion status from the real-time rehabilitation exercise and the video. The comprehension of our approach delineates the full range of motion into a systematic five-step process, commencing from the initial starting position to meticulous evaluation for exercise efficacy. This structured framework aims to facilitate home-based rehabilitation for patients, providing them with a clear understanding of their current condition and progression in exercises. The delineation of the full range of motion into discrete steps enhances patient comprehension and engagement in rehabilitation routines. By offering clear benchmarks of progress, individuals can readily discern their improvement trajectory. Additionally, this system empowers physiotherapists to effectively guide patients and enable tailored interventions to address specific needs. Furthermore, integrating this framework into tele-rehabilitation practices fosters seamless communication between healthcare providers and patients. By demarcating starting positions and providing real-time feedback on movement quality, patients can receive remote guidance with precision. Moreover, clinicians can assess both active and passive movements, facilitating comprehensive evaluation and monitoring of patient progress. Central to this model is its ability to quantify range of motion (ROM) through dynamic assessment of angles and movement status. This functionality empowers clinicians to gauge improvements and accurately track patient outcomes over time. Consequently, this innovative tool serves as a valuable resource for patients and healthcare professionals, aligning with the goal of optimizing rehabilitation outcomes through enhanced accessibility and precision.

**Algorithm 1:**
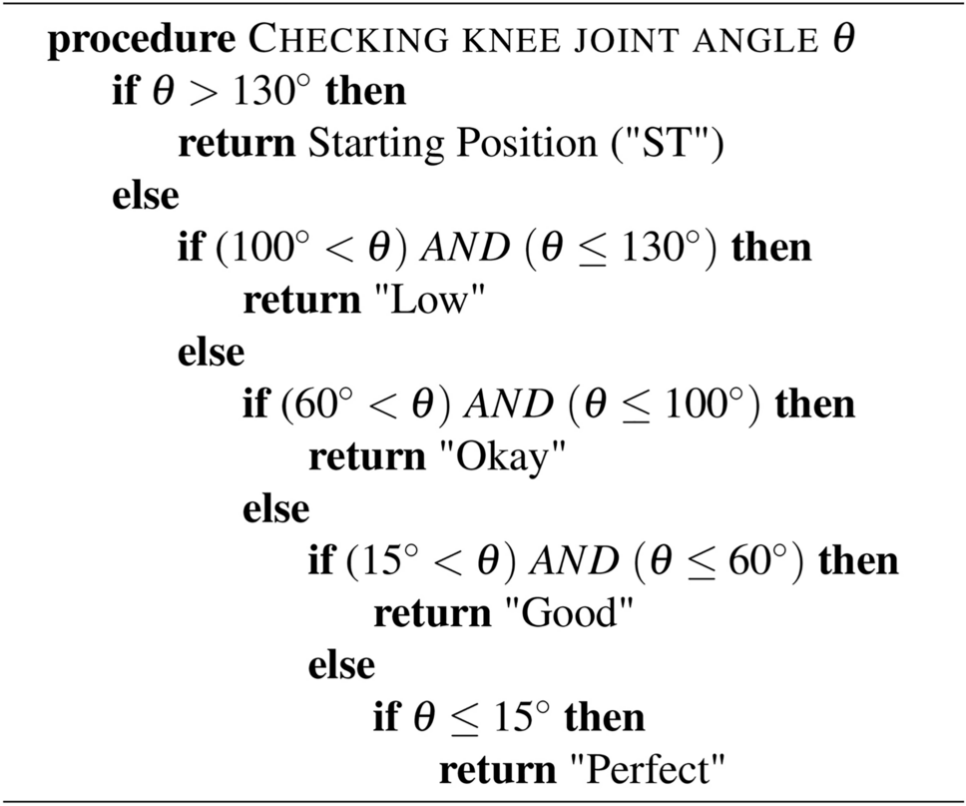
Hip Flexion Status.

**Fig. 16:**
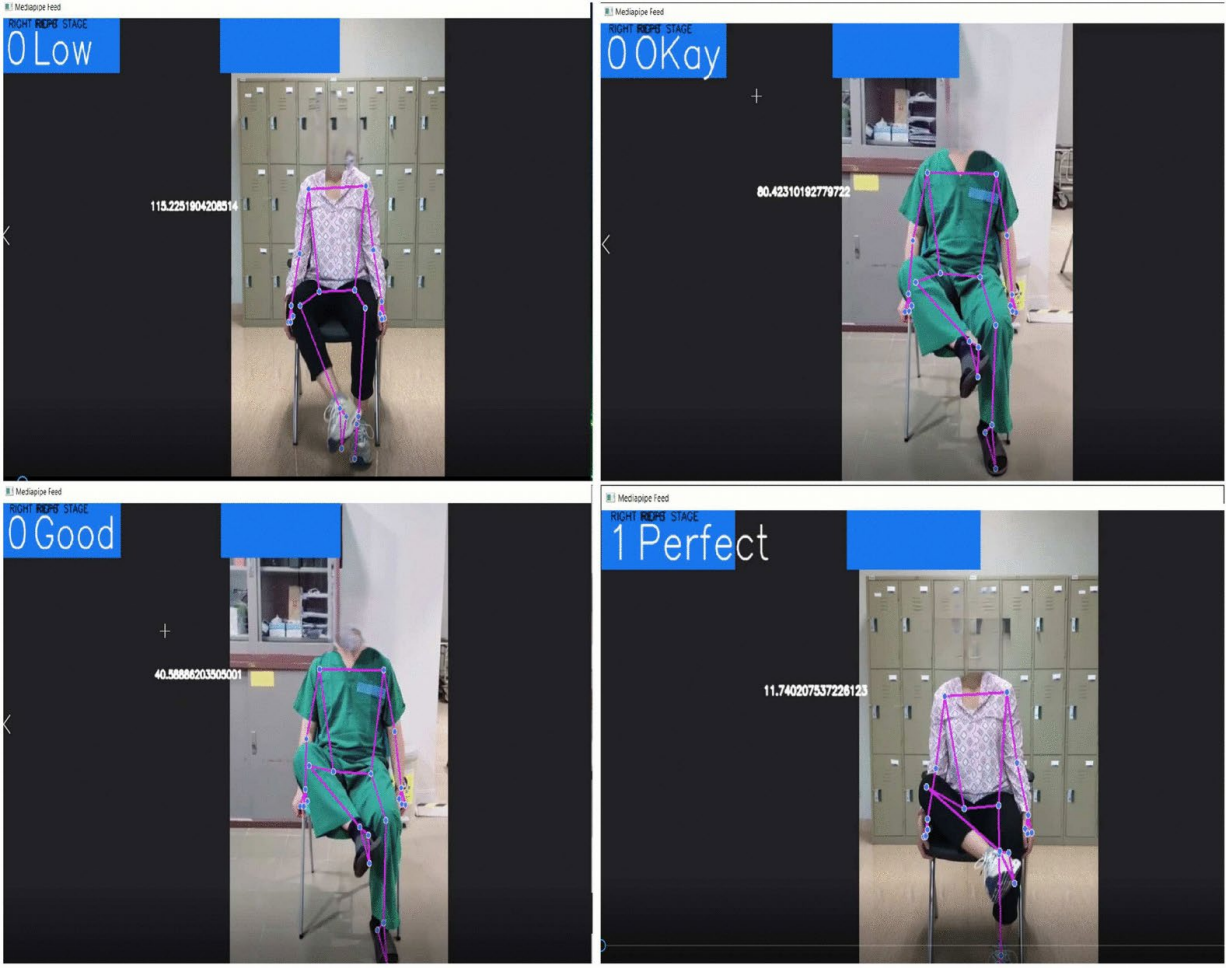
Four status results of hip external rotation exercise from real-time and recorded video.

For the hip external rotation, if $\theta$ is greater than $130^{\circ }$ it means the “Starting Position (ST)”; when it is less than or equal $130^{\circ }$ and greater than $100^{\circ }$, it reaches the “Low” status. In the range of less than or equal $100^{\circ }$ and greater than $60^{\circ }$ it reaches the status of “Okay”; within the range of greater than $30^{\circ }$ and less than or equal $60^{\circ }$ it is “Good” and last when it is less than or equal $30^{\circ }$ its status changes to “Perfect”. The primary objective of this approach is to encourage patients to actively work towards achieving the full range of motion, which may be limited by muscle weakness, spasticity, or other factors following a stroke. By starting at the end range of motion, patients are motivated to move towards the neutral position ($0^{\circ }$) and then beyond, ultimately improving their motor function and range of motion. Algorithm 2 summarizes the pseudo code of the status changing of hip external rotation exercise.

**Algorithm 2:**
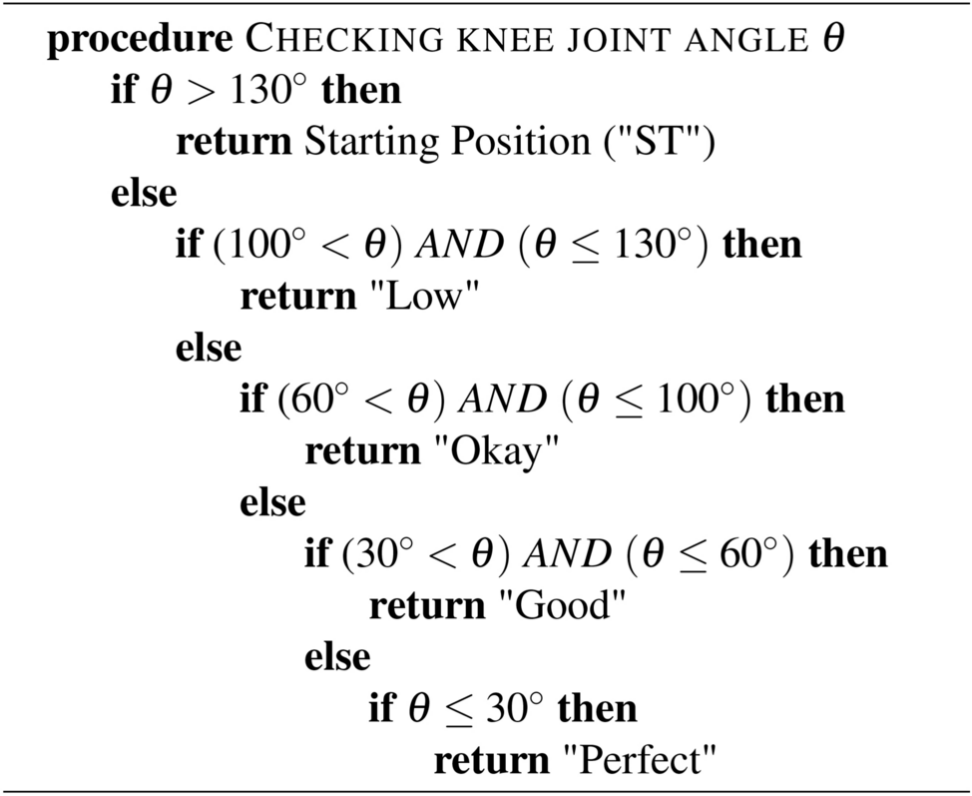
Hip External Rotation.

Figure 16 shows the status change of the hip external rotation exercise using both real-time and video based on the Algorithm 2. Finally, Table 4 summarizes all performed lower limb rehabilitation exercise types along with their degree of trajectory. It also provides the predicted degree of angle by our developed hybrid model and the actual angle measured through traditional mathematical calculation. Table 4 also includes the error between the actual and predicted values by our model.

### Angle error evaluation

Our model detects the point or landmark of a body joint, and we extract the point position from recorded or real-time video. The joint point is the floating number; the point value changes when a particular body part moves. During the activity, it is not a fixed point but a floating point. It needs to be calculated using three other joint positions for the particular joint angle. Based on the past frame, assume the next frame. When we save some image frames and compare them with the geometrical model, there are differences between this ML-Driven model representation and our geometrical, mathematical model output. So, we calculate the difference between the actual and predicted data as below.

$$\begin{aligned} \text {Mean Absolute Error (MAE)}= & \frac{\sum |y_i - \hat{y}|}{N} \\ \\ \text {MAE}= & 0.53 \\ \\ \text {Mean Squared Error (MSE)}= & \frac{\sum (y_i - \hat{y})^2}{N} \\ \text {MSE}= & 7.8135 \\ \text {Root Mean Squared Error (RMSE)}= & \sqrt{\frac{1}{N} \sum _{i=1}^N(y_i-\hat{y})^2} \\ \text {RMSE}= & 2.7953 \end{aligned}$$

8 $$\begin{aligned} \text{( }Coefficient~of~determination) R^2 = 1 - \frac{\sum (y - \hat{y})^2}{\sum (y_i - \widetilde{y})^2} \end{aligned}$$

$$\begin{aligned} R^2 = 0.99869 \end{aligned}$$

$R^2$ is the coefficient of determination. It is a statistical metric used to evaluate the efficacy of the fit of a regression model. *y* represents the observed values of the dependent variable. $\hat{y}$ represents the predicted values of the dependent variable generated by the regression model. ${y_i}$ represents individual observed values from the dataset. The numerator ${\sum (y-\hat{y})^2}$ represents the sum of squared residuals, which measures the discrepancy between the observed values and the predicted values. The denominator ${\sum \left( y_i-\widetilde{y}\right) ^2}$ represents the total sum of squares, where $\widetilde{y}$ is the mean of the observed values. It measures the total variance in the dependent variable. The coefficient of determination $R^2$ is then calculated as 1 minus the ratio of the sum of squared residuals to the total sum of squares. This ratio represents the proportion of the total variance in the dependent variable that the regression model explains. An $R^2$ value of 1 indicates that the model is a perfect fit, where all the variance in the dependent variable is explained by the independent variable(s). An $R^2$ value of 0 indicates no relationship between the independent and dependent variables. The proposed model $R^2$ value is 0.99869, which means the proposed model is very close to perfectly fitting.

## Discussion

The experimental findings indicate that our proposed model represents a promising approach to stroke rehabilitation, applicable in both clinical and tele-rehabilitation settings. By incorporating biofeedback techniques with task-oriented training, the model can enhance stroke patients’ motor function, range of motion, and functional outcomes. Notably, using a starting position of 180$^\circ$ instead of the traditional zero degrees may motivate patients to work towards the full range of motion, ultimately improving their motor function. Furthermore, the hybrid approach enables real-time feedback on movement patterns, which can help reinforce correct movements and discourage compensatory strategies. The validity of these findings is corroborated by the low error rates observed in the developed hybrid model, with an MAE of 0.53, an MSE of 7.8135, and an RMSE of 2.7953, indicating relatively accurate predictions. These results suggest that the hybrid biofeedback model holds the potential to provide an effective and reliable means of improving motor function in stroke patients. However, further verification through medical imaging, such as MRI or CT scans, may be necessary to assess the full extent of patient improvement, given the long-term nature of post-stroke rehabilitation.

The proposed ML-driven rehabilitation model offers several advantages over traditional approaches. First, the integration of multiple ML techniques, including CNN, fuzzy logic, and K-NN, allows for a more comprehensive and adaptive rehabilitation program that can respond to the unique needs and progress of each patient. The convolutional neural network component enables accurate real-time tracking and analysis of patient movements, allowing the system to provide personalized feedback and adjust the rehabilitation exercises accordingly. The fuzzy logic system, on the other hand, enables the model to handle the inherent uncertainties and complexities associated with human movement, ensuring a more nuanced and personalized approach to rehabilitation.

Comprehensive lower limb functionality assessments before and after interventions are crucial in post-stroke rehabilitation. Physicians thoroughly review the patient’s medical history, considering factors such as stroke type, severity, comorbidities, and previous mobility levels. Evaluations involve muscle testing, dynamometry, and range-of-motion assessments of affected joints, including the hip, knee, and ankle. Additionally, muscle tone, reflexes, and sensory deficits in the lower limbs are analyzed. Standardized methods are employed to evaluate hip flexion, knee extension, and hip external rotation, assessing coordination and balance. A follow-up physical exam emphasizes these same parameters. By comparing post-intervention measurements of muscle strength, range of motion, coordination, balance, and gait with baseline data, progress can be gauged. Patient feedback is gathered, and rehabilitation goals are evaluated for modification. The treatment plan is then updated based on the post-intervention assessment. The K-NN-based model enables the visualization and comparison of movement patterns, while the hybrid model facilitates the acquisition of desired angles and the Fugl-Meyer Assessment, enabling the assessment of improvements in angles and range of motion.

**Fig. 17:**
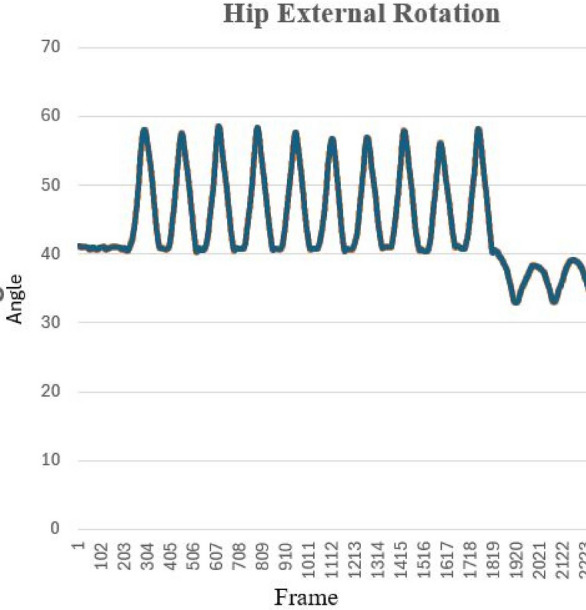
Visualization of angle result for the hip external rotation exercise.

**Fig. 18:**
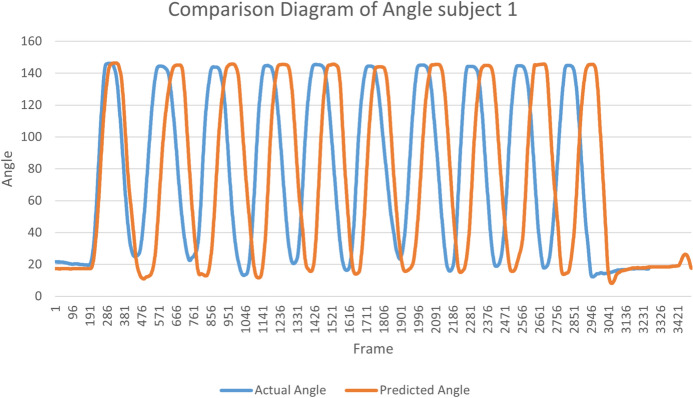
Comparison chart between predicted angle value angles and actual angles of elbow flexion exercise.

**Fig. 19:**
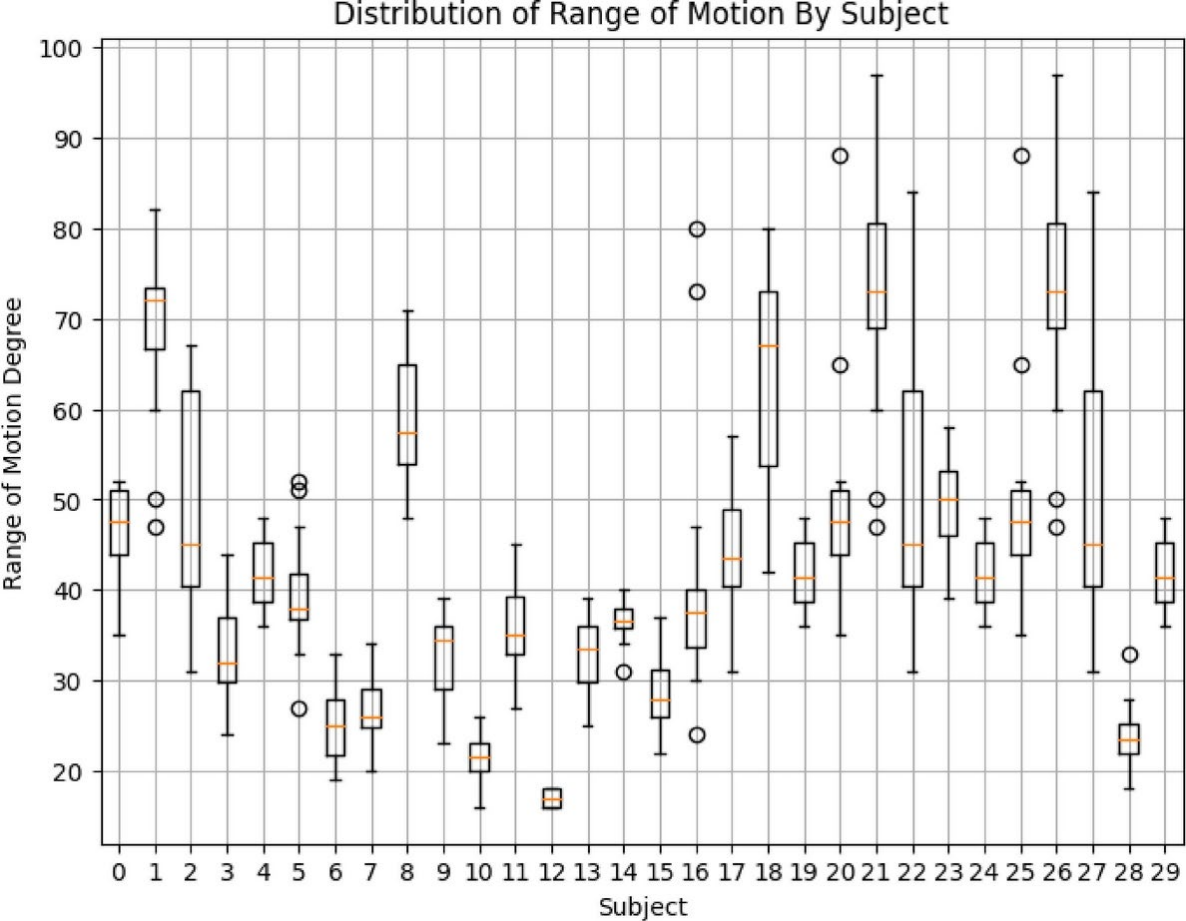
The output result of the graphical visualization for the hip external rotation of 30 subjects.

The ML-Driven model enables the generation of graphical visualizations from the video data collected. Typically, healthcare providers recommend ten repetitions of an exercise. Figure 17 depicts the range of motion observed for a subject’s hip external rotation, which fluctuated between $16^\circ$ and $18^\circ$, with an average of $17^\circ$, over the course of ten repetitions. Figure 18 presents a comparison chart of the changes over time between predicted posture angles and the actual measured angles of elbow flexion exercise. The data is recorded on a frame-by-frame basis according to the performance time. This model allows for the simultaneous visualization of the subject’s performance across multiple exercises, facilitating comparative analyses to assess patient progress over time. Healthcare professionals can leverage tele-rehabilitation platforms to access and interpret these visualizations, enabling remote monitoring and intervention. Furthermore, Fig. 19 illustrates the hip external rotation range of motion for a cohort of 30 persons, showcasing the variability between individuals. The total number of repetitions observed was 600, with each subject completing 10 repetitions for each leg. To validate the reliability of our model, we performed the Intraclass Correlation Coefficient analysis on the range of motion data. The ICC study results were excellent, with a score of about 1.0.

**Fig. 20:**
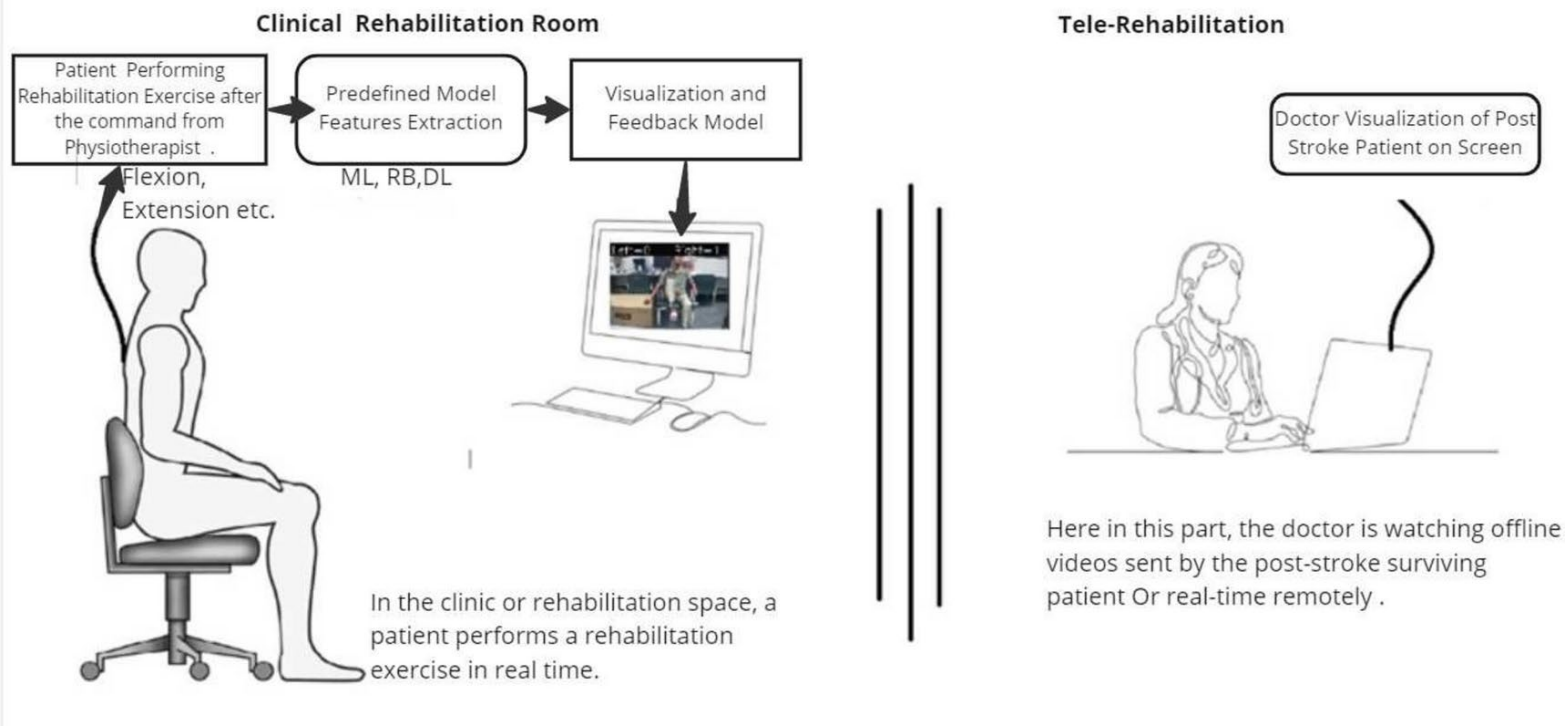
Proposed practical clinical environment for tele-rehabilitation using our proposed ML-Driven approach.

Despite the meticulous design of this approach, several challenges and concerns merit consideration. Firstly, the reliance on a single camera necessitates precise camera placement and patient positioning adjustments prior to the experiment. Vigilance is crucial to minimize inadvertent interruptions, such as individuals crossing in front of the camera, given the model’s focus on tracking a single person. Technical issues, including hardware malfunctions, software glitches, or connectivity problems, may potentially disrupt the data collection process. Moreover, the intervention’s dependence on equipment or resources could impede accessibility, particularly in settings with limited resources or during home-based rehabilitation. Clinicians must undergo training to proficiently interpret the ML model output and effectively integrate it into their clinical decision-making. Leveraging an ML model for objectively measuring range of motion offers advantages, such as standardized and quantifiable data, which facilitates precise progress tracking over time. Objective measurements also foster improved communication among healthcare professionals and enhance the reproducibility of assessments. Furthermore, our ML-Driven model holds promise in providing real-time feedback to patients and clinicians during exercise sessions, enabling the correction of techniques and optimizing performance as shown in Fig. 20. This immediate feedback mechanism can augment motivation and engagement in rehabilitation exercises, ultimately enhancing the overall efficacy of the intervention.

## Conclusion

This study developed an integrated approach of two models for tracking lower limb exercises of stroke patients. The first model is a novel fuzzy logic-based rule system that accurately counts exercises and extracts joint angles. It provides visual feedback to motivate patients to perform exercises correctly. This model also assists clinicians in visualizing the patient’s range of motion. The second model utilizes a K-Nearest Neighbors algorithm to graphically evaluate and quantify the patient’s post-stroke exercises. The proposed models address a gap in the literature, as no previous ML-driven rehabilitation approaches exist. The goal is to create ML-based solutions to support clinicians in hospitals and enable home-based or tele-rehabilitation for patients. Additionally, the models can aid physicians in patient diagnosis and determining optimal rehabilitation dosages.

Experimental results demonstrated the effectiveness of the models. The K-NN model achieved high accuracy in tracking hip flexion ($97\%$), hip external rotation ($92\%$), and knee extension ($91\%$) for a group of 30 physical therapists at King Chulalongkorn Memorial Hospital, Bangkok, Thailand. The rule-based model showed strong correlation ($R^{2} = 0.99869$) with mathematical calculations, with low error metrics ($MAE = 0.53$, $MSE = 7.8135$, $RMSE = 2.7953$). These findings suggest the proposed models can effectively support post-stroke and home-based rehabilitation.

Further research is needed to validate the results and optimize the hybrid biofeedback model. While this study focused on lower limb rehabilitation, future work should explore the model applicability to upper limb impairments and various exercise regimens. Lastly, longitudinal patient studies are required to assess the long-term viability and real-world impact of these ML-driven rehabilitation systems.

## Supplementary Information

Supplementary Information.

## Data Availability

The data will be available upon request due to ethical issues. Please contact the corresponding author at timporn.v@chula.ac.th to request the data.
