# Supplementary material for: An innovative model based on machine learning and fuzzy logic for tracking lower limb exercises in stroke patients
Source: Sci Rep. 2025 Apr 2;15:11220. doi: 10.1038/s41598-025-90031-1 (PMC11965402; doi:10.1038/s41598-025-90031-1)
Supplement: Supplementary file 1 — Supplementary Information. [file 41598_2025_90031_MOESM1_ESM.pdf]

*Appendices A

Paper: AI-Driven Rehabilitation: Integrating CNN, Fuzzy Logic, and K-NN Models for Lower Limb Recovery in Stroke Patients

Human Subjects Data used in this paper

Subject No	Ages	Gender	Weight (kg)	Underlying	Hip Flexion Performing Time (Second)	Hip External Rotation Performing Time (Second)	Knee Extension performing time
1	34	F	54	no	94	86	98
2	31	F	43	no	43	41	53
3	35	M	70	Hepatitis B	46	47	48
4	21	M	65	no	49	48	49
5	25	F	45	no	33	32	31
6	27	M	60	no	47	38	51
7	49	F	93	Diabetes	48	55	51
8	28	F	51	no	52	56	61
9	30	M	95	no	44	48	48
10	36	M	67	Fatty liver	47	46	50
11	39	M	74	No	39	42	48
12	27	M	57	No	82	78	80
13	33	F	45	No	47	40	47
14	31	M	75	No	50	57	58
15	39	F	78	No	30	30	41
16	31	M	96	No	37	35	42
17	31	M	78	No	45	40	44
18	37	M	68	No	40	42	39
19	34	M	72	No	42	45	41
20	24	F	75	No	36	40	53
21	22	F	76	No	48	57	58
22	22	F	63	No	39	43	54
23	20	F	57	No	43	47	52
24	22	F	60	No	62	60	67
25	21	F	45	No	48	34	41
26	21	F	50	No	36	37	50

27	22	F	50	No	61	65	62
28	28	M	96	No	50	54	45
29	25	M	55	No	40	44	41
30	22	M	62	No	41	40	46

Ethics approval and consent to participate

The Institutional Review Board of the Faculty of Medicine, Chulalongkorn University, Bangkok, Thailand, has approved the following study in compliance with the international guidelines for human research protection as Declaration of Helsinki and its subsequent amendments, The Belmont Report, CIOMS Guideline and International Conference on Harmonization in Good Clinical Practice (ICH-GCP)

Data:
The total number of subjects who participated in this research is 30. Among them, 15 males and 15 females. The participant subjects age range is (19 -50). Among them, subject 3 has Hepatitis B disease, subject 7 has diabetes, and subject 10 has a fatty liver. In the case of these 3 subjects, we didn’t find any abnormality during the exercise. All the subjects perform each exercise for the 10 repetitions.
